# Comparative analysis of retroviral Gag-host cell interactions: focus on the nuclear interactome

**DOI:** 10.1186/s12977-024-00645-y

**Published:** 2024-06-19

**Authors:** Gregory S. Lambert, Breanna L. Rice, Rebecca J. Kaddis Maldonado, Jordan Chang, Leslie J. Parent

**Affiliations:** 1 Department of Medicine, Penn State College of Medicine, 500 University Drive, Hershey, PA 17033 USA; 2 Department of Microbiology and Immunology, Penn State College of Medicine, 500 University Drive, Hershey, PA 17033 USA

**Keywords:** Retroviruses, Rous sarcoma virus, HIV-1, Mass spectrometry, Proteomics

## Abstract

Retroviruses exploit host proteins to assemble and release virions from infected cells. Previously, most studies focused on interacting partners of retroviral Gag proteins that localize to the cytoplasm or plasma membrane. Given that several full-length Gag proteins have been found in the nucleus, identifying the Gag-nuclear interactome has high potential for novel findings involving previously unknown host processes. Here we systematically compared nuclear factors identified in published HIV-1 proteomic studies and performed our own mass spectrometry analysis using affinity-tagged HIV-1 and RSV Gag proteins mixed with nuclear extracts. We identified 57 nuclear proteins in common between HIV-1 and RSV Gag, and a set of nuclear proteins present in our analysis and ≥ 1 of the published HIV-1 datasets. Many proteins were associated with nuclear processes which could have functional consequences for viral replication, including transcription initiation/elongation/termination, RNA processing, splicing, and chromatin remodeling. Examples include facilitating chromatin remodeling to expose the integrated provirus, promoting expression of viral genes, repressing the transcription of antagonistic cellular genes, preventing splicing of viral RNA, altering splicing of cellular RNAs, or influencing viral or host RNA folding or RNA nuclear export. Many proteins in our pulldowns common to RSV and HIV-1 Gag are critical for transcription, including PolR2B, the second largest subunit of RNA polymerase II (RNAPII), and LEO1, a PAF1C complex member that regulates transcriptional elongation, supporting the possibility that Gag influences the host transcription profile to aid the virus. Through the interaction of RSV and HIV-1 Gag with splicing-related proteins CBLL1, HNRNPH3, TRA2B, PTBP1 and U2AF1, we speculate that Gag could enhance unspliced viral RNA production for translation and packaging. To validate one putative hit, we demonstrated an interaction of RSV Gag with Mediator complex member Med26, required for RNA polymerase II-mediated transcription. Although 57 host proteins interacted with both Gag proteins, unique host proteins belonging to each interactome dataset were identified. These results provide a strong premise for future functional studies to investigate roles for these nuclear host factors that may have shared functions in the biology of both retroviruses, as well as functions specific to RSV and HIV-1, given their distinctive hosts and molecular pathology.

**Graphical Abstract:**

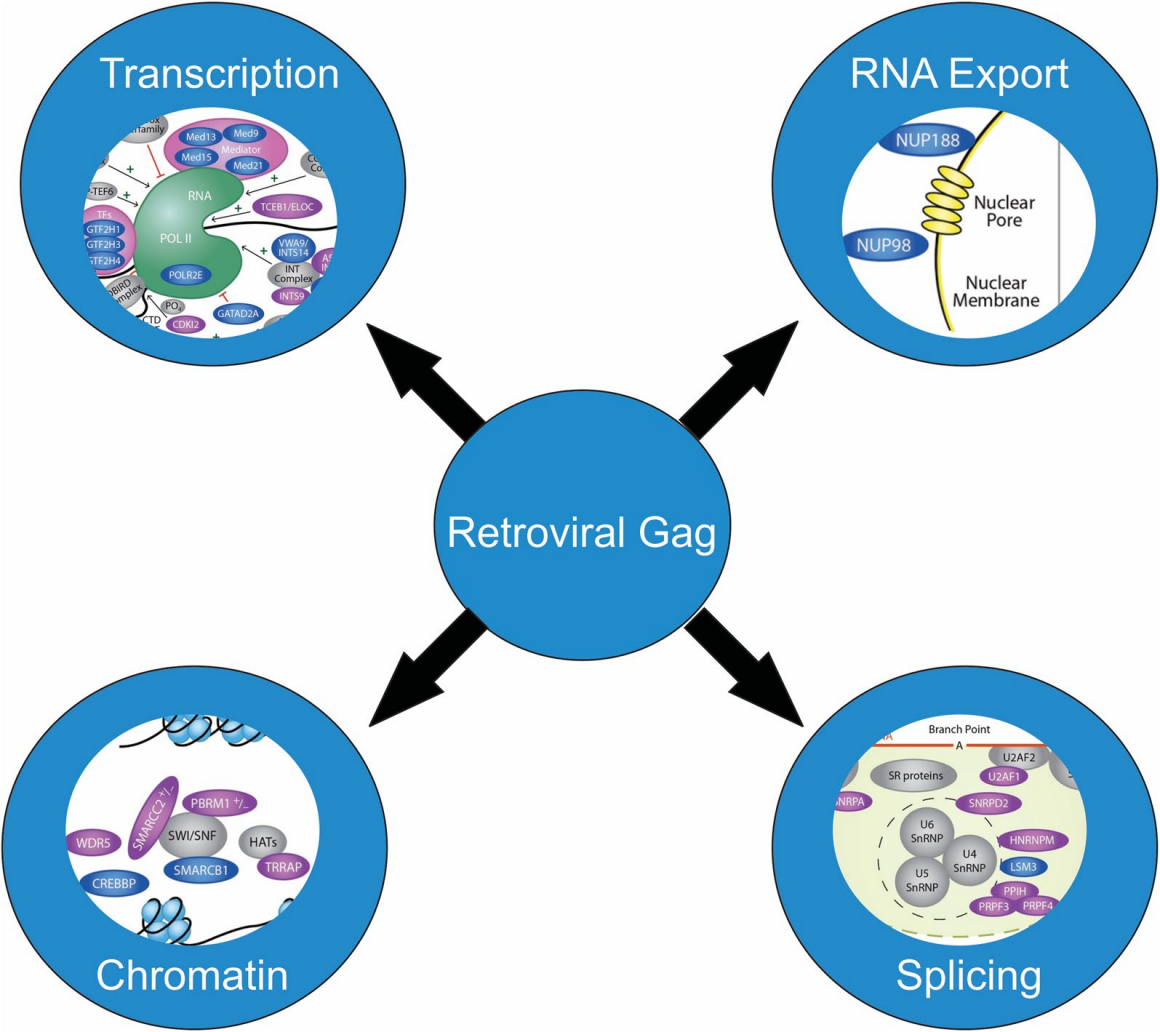

**Supplementary Information:**

The online version contains supplementary material available at 10.1186/s12977-024-00645-y.

## Introduction

Retroviral replication depends upon the selection and packaging of unspliced viral RNA (USvRNA) as the genome by the Gag polyprotein, the major structural protein shared by retroviruses. Historically, it was thought that the Gag protein performed this function in the cytoplasm before trafficking to the plasma membrane where budding of virions occurs. However, there is now compelling evidence that a population of retroviral Gag proteins enter the nucleus where they may initiate selection of genomic RNA (gRNA). The strongest evidence for this paradigm shift is based on studies of the Rous sarcoma virus (RSV) Gag protein, which depends on transient nucleocytoplasmic trafficking facilitated by host transport proteins to ensure efficient gRNA packaging [1–6]. This requirement for nuclear trafficking was demonstrated by a mutant of Gag that bypassed the nucleus, resulting in decreased gRNA packaging which was increased with restoration of nuclear localization [7]. RSV Gag co-opts the host karyopherins importin α/β, importin-11, and transportin-3 (TNPO3) to enter the nucleus [1–3, 6], and nuclear egress is mediated by binding of a hydrophobic nuclear export signal (NES) in the p10 domain to the CRM1-RanGTP nuclear export complex [3–5]. A more recent study demonstrated that RSV Gag binds to newly synthesized USvRNA in discrete ribonucleoprotein complexes (RNPs) in the nucleus, and these Gag-vRNA RNPs have been observed trafficking across the nuclear envelope into the cytoplasm [8].

A population of human immunodeficiency virus type-1 (HIV-1) Gag, like that of RSV Gag, also undergoes nuclear localization [9–13]. HIV-1 Gag forms focal RNP complexes with nascent USvRNA in the nucleus, and traffics to the major viral RNA transcription site in T cells reactivated from latency [9]. Nuclear localization of HIV-1 Gag occurs in a concentration-independent manner shortly after Gag synthesis begins, and Gag colocalizes with transcriptionally-active euchromatin near the nuclear periphery [10]. The function(s) of these nuclear RNPs has yet to be thoroughly investigated, although these data demonstrate that both RSV and HIV-1 Gag proteins traffic to transcription sites and associate with their cognate USvRNAs. Although one possibility is that Gag-USvRNA binding in the nucleus could initiate the genomic RNA packaging process, nuclear localization of Gag could also influence other important cellular processes, such as regulation of viral or host transcription, RNA modification or processing, splicing, chromatin remodeling, or RNA export.

In addition to RSV and HIV-1, the Gag proteins of other retroviruses, including murine leukemia virus (MLV), prototype foamy virus (PFV), feline immunodeficiency virus (FIV), mouse mammary tumor virus (MMTV), and Mason-Pfizer monkey virus (MPMV) also have been shown to localize to the nucleus [11, 14–27]. For example, the PFV Gag protein is involved in proviral integration through its interaction with chromatin [27]. The finding that nuclear trafficking of Gag is a feature conserved among many retroviruses raises the likelihood that nuclear-localized Gag proteins participate in functions important for virus replication. There are also Gag cleavage products that undergo nuclear localization, including the nucleocapsid (NC) proteins of HIV-1, RSV, MLV, and MMTV, which localize to the nucleolus [11, 19, 22, 28], and the p12 protein of MLV, which binds to chromatin and influences proviral integration [23, 29].

It is well known that retroviruses exploit a variety of host pathways during replication, but previous investigation of host factors that bind to Gag have focused on factors localized to the cytoplasm and plasma membrane. However, the nuclear localization of retroviral Gag proteins raises important questions concerning their functions, which can be informed by identifying nuclear host partners. To gain further insight into what nuclear processes Gag could be influencing, we comprehensively analyzed and systematically compared six previously published HIV-1 proteomic studies performed by other laboratories, which used various experimental approaches to identify novel host proteins that interact with HIV-1 Gag. A variety of techniques are represented in this analysis, including affinity purifications of GFP-tagged Gag, tandem affinity purification of Gag, and BirA* Gag complexes [30–35]. To complement those datasets, we performed affinity-tagged purification of both RSV and HIV-1 Gag, and identified nuclear interacting partners using mass spectrometry. To further explore one of the novel hits, we utilized immunoprecipitation and quantitative imaging approaches to validate the interaction of RSV Gag with Mediator complex subunit 26 (Med26; AlphaFold Protein Structure Database [36, 37], entry O95402), a critical component of the transcriptional Mediator complex, which is exploited by other viruses and endogenous retroelements [38–45]. Together, published studies combined with our results suggest that Gag proteins may interface with host nuclear factors to facilitate genomic RNA selection and/or influence cellular processes, including gene expression, RNA processing, splicing, nucleic acid metabolism, and/or chromatin modification.

## Materials and methods

### Cells, plasmids, and purified proteins

DF1 chicken embryo fibroblast cells, HeLa human cervical cancer cells, and QT6 quail fibroblast cells were maintained as described [11, 46, 47]. The RSV Gag expression constructs pGag.ΔPR (referred to herein as RSV Gag), pGag.L219A.ΔPR (referred to herein as RSV Gag.L219A), pGag.ΔNC, and pGag.ΔPR-GFP (referred to herein as RSV Gag-GFP) [4, 11, 48] and plasmids encoding for *Escherichia coli* (*E. coli*) expression of His-tagged RSV Gag (pET28.TEV-Gag.3 h) and HIV Gag (pET28a.WT.HIV.Gag.Δp6) were previously described [49, 50].

### Subcellular fractionation

QT6 cells were transfected with untagged RSV Gag constructs using the calcium phosphate method [51]. Sixteen hours later, the medium was changed to fresh primary growth medium (PGM) and the cells were allowed to recover for 24 h. All subsequent steps were performed on ice or at 4 °C with cold buffers unless otherwise stated. Cells were fractionated using the method described in [52] with some minor modifications, as below. Cells were removed from the plates using trypsin and then washed in cold PBS. The cell pellet was resuspended in sucrose buffer (10 mM HEPES pH 7.9, 10 mM KCl, 2 mM magnesium acetate, 3 mM CaCl_2,_ 340 mM sucrose, 1 mM DTT, 100 μg/ml phenylmethanesulfonyl fluoride (PMSF), 1 μg/ml pepstatin, and Roche Complete Protease Inhibitor Cocktail) and incubated on ice for 10 min. IGEPAL Nonidet P-40 was added to the final concentration of 0.5% and cells were vortexed on high for 15 s, and then spun for 10 min at 3,500*g* at 4 °C. The supernatant (cytoplasm fraction) was collected, and the pelleted nuclei were resuspended in nucleoplasm extraction buffer (50 mM HEPES pH 7.9, 150 mM potassium acetate, 1.5 mM MgCl_2,_ 0.1% IGEPAL Nonidet P-40, 1 mM DTT, 100 μg/ml PMSF, 1 μg/ml pepstatin, and Roche Complete Protease Inhibitor Cocktail) and transferred to a Dounce homogenizer and homogenized with 20 slow strokes. The homogenates were checked under a light microscope for completion of nuclear lysis, then transferred to a new tube and rotated at 4 °C for 20 min. The lysates were spun at 16,000*g* for 10 min at 4 °C. The supernatant (nucleoplasmic fraction) was collected and the remaining chromatin-containing pellet was resuspended in nuclease incubation buffer (50 mM HEPES pH 7.9, 10 mM NaCl, 1.5 mM MgCl_2_, 1 mM DTT, 100 μg/ml PMSF, 1 μg/ml pepstatin, and Roche Complete Protease Inhibitor Cocktail) with 100 U/ml of OmniCleave nuclease (Epicentre) for 10 min at 37 °C. NaCl was added to a final concentration of 150 mM and the lysates were incubated on ice for 20 min and spun for 10 min at 16,000*g* at 4 °C. The supernatant (low-salt chromatin fraction) was collected and the pellet was resuspended in chromatin extraction buffer (50 mM HEPES pH 7.9, 500 mM NaCl, 1.5 mM MgCl_2_, 0.1% Triton X-100, 1 mM DTT, 100 μg/ml PMSF, 1 μg/ml pepstatin, and Roche Complete Protease Inhibitor Cocktail), incubated for 20 min on ice, spun for 10 min at 16,000*g* at 4 °C, and the supernatant (high-salt chromatin fraction) was collected.

### Western blot analysis of subcellular fractions

Proteins from the subcellular fractions were analyzed via SDS-PAGE. Aliquots of the fractions were heated to 90 °C in 4X SDS-PAGE sample buffer (250 mM Tris–HCl, pH 6.8, 40% glycerol, 0.4% bromophenol blue, 8% SDS, and 8% β-mercaptoethanol) for 10 min prior to loading on a 10% SDS-PAGE gel and analyzed by western blot. Proteins were detected using antibodies against RSV Gag [53], Calnexin (Enzo Life Sciences ADI-SPA-865), Med4 (Abcam ab129170), RCC1 (Abcam ab54600), Histone H2B (Abcam ab52484), GAPDH (UBP Bio Y1040), and the appropriate HRP-conjugated secondary antibodies (Invitrogen).

Signal densities of the protein bands on the antibody-stained membranes were analyzed using Bio-Rad Image Lab Software on a ChemiDoc MP system. Rectangles were drawn around each band, as well as a blank background region, using the volume tools feature to quantify the signal intensity of each band. The background subtraction method was set to local, and the blank region that was highlighted by a rectangle was labelled as the background volume. The volumes report table was exported to Microsoft Excel. For each band corresponding to the Gag signal, the adjusted volumes for each fraction were added together to calculate the total adjusted volume. Then the percentages of each fraction were calculated by subtracting the fraction’s adjusted volume from the total adjusted volume. Averages and standard deviations were calculated for each fraction for each Gag protein from three separate experiments.

### Purified RSV Gag and HIV Gag pulldowns

#### Lysate preparation

DF1 and HeLa cells were fractionated using the NE-PER Nuclear and Cytoplasmic Extraction kit (ThermoFisher Scientific). All steps and buffers used were performed on ice or at 4 °C unless otherwise stated. Cells were lysed in CERI buffer containing the Complete Protease Inhibitor Cocktail (Roche). Cells were vortexed on the highest setting for 15 s and incubated on ice for 10 min. Ice-cold CERII buffer was added and cells were vortexed on high for 5 s then centrifuged for 5 min at 16,000*g* in a microcentrifuge. The supernatant was collected (cytoplasmic fraction), and the pelleted nuclei were resuspended in ice-cold NER buffer with protease inhibitor cocktail added. The nuclei were vortexed on high for 15 s and incubated on ice for 10 min, then vortexed for 15 s every 10 min for a total of 40 min. The lysed nuclei were centrifuged at 16,000*g* for 10 min. The supernatant (nuclear fractionation) was diluted to 14 ml with Buffer A (25 mM Tris–HCl pH 8.0, 200 mM NaCl, 2 mM 2-Mercaptoethanol (BME), and protease inhibitor cocktail). The nuclear fraction was concentrated to ~ 1 ml in a 3 kD MWCO Amicon column and then was diluted to 14 ml and concentrated once more to ~ 1.2 ml.

#### Nickel affinity purifications

Three reactions were performed using 6 μg of RSV H6.Gag.3 h or HIV-1 WT.Gag.Δp6.H6, and a no protein control for DF1 and HeLa nuclear lysates, respectively. The proteins and no protein control were incubated with pre-washed nickel beads for 1 h at 4 °C with rotation. The beads were then washed three times in Wash Buffer (300 mM NaCl, 50 mM NaH_2_PO_4_, pH 8.0), followed by incubation with 500 μg of nuclear extract for 2 h at 4 °C with rotation. The beads were washed again three times in Wash Buffer, and bound proteins were eluted from the beads using Wash Buffer + 300 mM imidazole for 15 min while rotating at 4 °C. The eluates were buffer exchanged into water using Zeba Spin Desalting Columns (ThermoFisher Scientific) and 20 ug of each sample was used for mass spectrometry analysis.

#### Sample preparation for mass spectrometry

The samples were prepared and processed at the Mass Spectrometry and Proteomics Core Research Facility at Penn State College of Medicine using an ABSciex 5600 TripleTOF. In a final volume of 100 μl, the samples were incubated in 50 mM NH_4_HCO_3_, pH 8.0, 10% v/v acetronitrile, and 0.1 μg trypsin for at least 3 h at 48 °C. To evaporate off the NH_4_HCO_3_ and acetronitrile, samples were dried down using a SpeedVac, and then resuspended in 200 μl H_2_O with vortexing. The drying was repeated 3X total, but the final resuspension volume was 10 μl. To each sample, a 1/9th volume of 1% formic acid was added.

### Mass spectrometry

The following mass spectrometry workflows were performed two separate times and data from both instances were combined to create the set of interactors presented herein.

#### 2D-LC Separations

SCX (strong cation-exchange) separations were performed on a passivated Waters 600E HPLC system, using a 4.6 X 250 mm PolySULFOETHYL Aspartamide column (PolyLC, Columbia, MD) at a flow rate of 1 ml/min. The gradient was 100% Buffer A (10 mM ammonium formate, pH 2.7, in 20% acetonitrile/80% water) (0–22 min following sample injection), 0% → 40% Buffer B (666 mM ammonium formate, pH 2.7, in 20% acetonitrile/80% water) (16–48 min), 40% → 100% Buffer B (48–49 min), isocratic 100% Buffer B (49–56 min), then at 56 min switched back to 100% Buffer A to re-equilibrate for the next injection. One milliliter fractions were collected and were dried down then resuspended in 9 µl of 2% (v/v) acetonitrile, 0.1% (v/v) formic acid, and were filtered prior to reverse phase C18 nanoflow-LC separation.

#### Mass spectrometry analysis

Each SCX fraction was analyzed following a calibration run using trypsin-digested β-Gal as a calibrant, then a blank run using the ABSciex 5600 TripleTOF. MS Spectra were then acquired from each sample using the newly updated default calibration, using a 60-min gradient from an Eksigent NanoLC-Ultra-2D Plus and Eksigent cHiPLC Nanoflex through a 200 µm × 0.5 mm Chrom XP C18-CL 3 µm 120 Å Trap Column and elution through a 75 µm × 15 cm Chrom XP C18-CL 3 µm 120 Å Nano cHiPLC Column.

#### Protein identification and analysis

Protein identification and quantitation were performed using the Paragon algorithm as implemented in ProteinPilot 5.0 software (ProteinPilot 5.0, which contains the Paragon Algorithm 5.0.0.0, build 4632 from ABI/MDS-Sciex) [54]. Spectra were searched against *Homo sapien* or *Gallus gallus* RefSeq subsets (plus 389 common contaminants) of the NCBInr database concatenated with a reversed "decoy" version of itself. For the ProteinPilot analyses, the preset Thorough Identification Search settings were used, and identifications needed to have a ProteinPilot Unused Score > 1.3 (> 95% confidence interval) to be accepted. In addition, the only protein identifications (IDs) accepted were required to have a "Local False Discovery Rate" estimation of no higher than 5%, as calculated from the slope of the accumulated Decoy database hits by the PSPEP (Proteomics System Performance Evaluation Pipeline) [55]. Proteins that were labelled as contaminants or reversed were removed from the analysis. The mass spectrometry proteomics data have been deposited to the ProteomeXchange Consortium via the PRIDE [56] partner repository with the dataset identifier PXD048774.

### Analysis of proteomics

The Database for Annotation, Visualization, and Integrated Discovery (DAVID, version 6.8) [57, 58] was used to assign each protein to its cellular compartment(s) and biological process categories. Proteins were organized by their gene name for entries into DAVID and the *Homo sapiens* species database was used. Data presented in the tables were generated using the Gene Ontology GOTERM_BP_ALL to categorize proteins by their biological function, and GOTERM_CC_ALL to first identify the proteins present in the nucleus. Categories with a p-value of ≤ 0.05, as determined by modified Fisher’s Exact Test, were considered statistically overrepresented, and any redundant categories (same p-value and proteins) were removed.

The Bioinformatics and Evolutionary Genomics online comparison tool was used to generate the Venn diagram (http://bioinformatics.psb.ugent.be/webtools/Venn/). Ingenuity Pathway Analysis (IPA) (QIAGEN Inc., https://www.qiagenbioinformatics.com/products/ingenuitypathway-analysis) was performed to categorize the functions of the identified proteins. Core Analysis was performed on the gene IDs that could be mapped by IPA, as some gene IDs were not recognized by IPA, on each separate proteomic list. Under the Core Analysis, Expression analysis was selected; direct and indirect relationships were examined. No endogenous chemicals were included in the analysis. The filters that were used included: all molecule types and data sources; confidence = experimentally observed; species = human only; no tissues or cell lines or mutations were included. Only examined categories associated with molecular and cellular functions, as outlined by [59]. Additionally, protein interaction maps were generated using STRING consortium (https://string-db.org/) to visualize clusters of protein–protein interactions.

### Generation of RC.V8-infected QT6 nuclear lysates

QT6 cells were infected for 4 h with cell culture medium obtained from a separate culture of QT6 cells transfected with pRC.V8. Cells were fractionated using the method described in [52] with minor modifications, as described below. Cells were trypsinized, pelleted at low speed, and washed in cold PBS. The cell pellet was resuspended in lysis buffer (10 mM HEPES pH 7.9, 10 mM KCl, 0.1 mM EDTA, 0.3% Nonidet P-40, 1 mM DTT, 100 μg/ml phenylmethanesulfonyl fluoride (PMSF), 1 μg/ml pepstatin, 100 U/ml Omnicleave (Epicentre), and Roche Complete Protease Inhibitor Cocktail) and incubated on ice for 5 min. Cells were then spun for 5 min at 3000 rpm at 4 °C to pellet nuclei, and the supernatant (cytoplasmic fraction) was collected. The pelleted nuclei were washed once with lysis buffer, then resuspended in nuclear extract buffer (20 mM HEPES pH 7.9, 10 mM NaCl, 1 mM DTT, 100 μg/ml PMSF, 1 μg/ml pepstatin, 100 U/ml Omnicleave (Epicentre), and Roche Complete Protease Inhibitor Cocktail) and incubated at 37 °C in a water bath for 10 min. Nuclear lysate was placed on ice, and solution was brought to 400 mM NaCl and 1 mM EDTA, followed by vortexing on high for 15 s and 20 min of rotation at 4 °C. Debris was pelleted at 13,000 rpm for 10 min at 4 °C, and supernatant was transferred to a fresh tube (nuclear fraction). Protein concentration in lysates was determined by Bradford assay.

### RSV Gag-Med26 Co-immunoprecipitation

RC.V8-infected nuclear lysates (500 µg) were pre-incubated for 2 h with α-RSV-CA antibody (mouse α-RSV CA.A11, gift from Neil Christensen, Penn State College of Medicine) in low salt NET2 buffer (50 mM Tris pH 7.4, 150 mM NaCl, 0.05% Nonidet P-40; [60]) at 4 °C with rotation. With 1 h remaining, Pierce™ Protein G Magnetic Beads (60 µl of a 50% slurry per reaction) were washed 4 times with high salt NET2 buffer (50 mM Tris pH 7.4, 400 mM NaCl, 0.05% Nonidet P-40; [60]), then blocked with 5% w/v BSA in low salt NET2 buffer at 4 °C with rotation. At the end of the 2 h, blocking buffer was removed and beads were resuspended in low salt NET2 buffer. An equal amount (~ 30 µl) was added to each reaction, and tubes were rotated at 4 °C overnight.

After overnight incubation, buffer was removed and beads were washed 4 times with high salt NET2 buffer. Bound proteins were then eluted by boiling beads in 50 µl of 1X SDS-PAGE buffer for 10 min at 100 °C. Beads were pelleted at 13,000 rpm for 5 min, and supernatant was taken for analysis.

Samples were run on 10% SDS-PAGE gels, transferred to PVDF, blocked for 30 min with 5% Milk/0.1% TBS-Tween, and then incubated with primary antibody (rabbit α-Med26, Proteintech, 21,043–1-AP) in 0.5% Milk/0.1% TBS-Tween at 4 °C with rocking overnight. Membranes were washed 3 times for 5 min with 0.1% TBS-Tween, then incubated with secondary antibody (goat α-rabbit-HRP, Sigma A0545) for 1 h at room temperature. Washes were repeated, and membranes incubated with ECL 2 for 5 min. Western blots were imaged using a BioRad ChemiDoc MP imager. Blots were stripped and reprobed for RSV Gag using rabbit α-RSV-W [gift from John Wills, Penn State College of Medicine [53] and secondary antibody (goat α-rabbit-HRP, Sigma A0545)].

### Confocal imaging

QT6 cells were plated at a density of 0.5 × 10^6^ cells/well in 6-well tissue culture dishes containing #1.5 coverslips and were allowed to settle overnight. The following afternoon, wells were transfected with 500 ng of RSV Gag-GFP [11] and 125 ng of FLAG-tagged Med26 (a gift from Joan Conaway and Ronald Conaway [Addgene plasmid #15,367; http://n2t.net/addgene:15367; RRID: Addgene_15367) [61]] expression vectors. The following morning, cells were washed 2X quickly with warm PBS, and fixed in 3.7% formaldehyde in PBS for 10 min at RT. Slides were then washed 3X with PBS (5 min per wash), permeabilized for 15 min in 0.25% Triton X-100/PBS, washed 3X, and blocked for 30 min at RT in 10% BSA/PBS. Relevant slides were incubated for 2 h with mouse α-FLAG-M2 antibody (Sigma, F1804) in 3% BSA/PBS in a humid chamber at 37 °C followed by 3X washes. Secondary antibody staining with donkey α-mouse-AlexaFluor647 (AF647) was carried out for 2 h in 3% BSA/PBS in a humid chamber at 37 °C, followed by 3X washes. Slides were DAPI stained for 1 min at RT, mounted with ProLong™ Diamond (Life Technologies), and cured for 24 h. Slides were imaged on a Leica AOBS SP8 confocal microscope with a 63x/1.4 oil objective, with pinhole set to 1 airy unit and frame averaging set to four. FLAG-Med26-AF647 was excited using a white light laser (WLL) tuned to 647 nm at 2% laser power, and emission was detected via hybrid detector. RSV Gag-GFP was excited by WLL tuned to 488 nm at 3% laser power and detected via hybrid detector. DAPI was excited using a 405 nm diode laser at 8% power and detected using a photomultiplier tube.

Colocalization between RSV Gag and Med26 was assessed using Imaris image analysis software (Oxford Instruments). Briefly, images were Gaussian filtered and surfaces were generated around cell nuclei using the DAPI channel as reference. Masks were created for RSV Gag and Med26 signal within nuclear surfaces, and colocalization of this signal was assessed using the Imaris colocalization tool. Manders’ Overlap Coefficients were exported from Imaris and data was assessed for outliers by Grubbs’ test with an α = 0.05 using GraphPad Prism (GraphPad Software, Inc.). Statistical analysis and generation of Fig. 3C was also done with this software. A total of 17 M1 (Med26 ∩ Gag) and 18 M2 (Gag ∩ Med26) individual data points were plotted. Representative images (Fig. 3B) and video (Video S1) were created using Imaris software.

## Results

### Subcellular localization

**Fig. 1:**
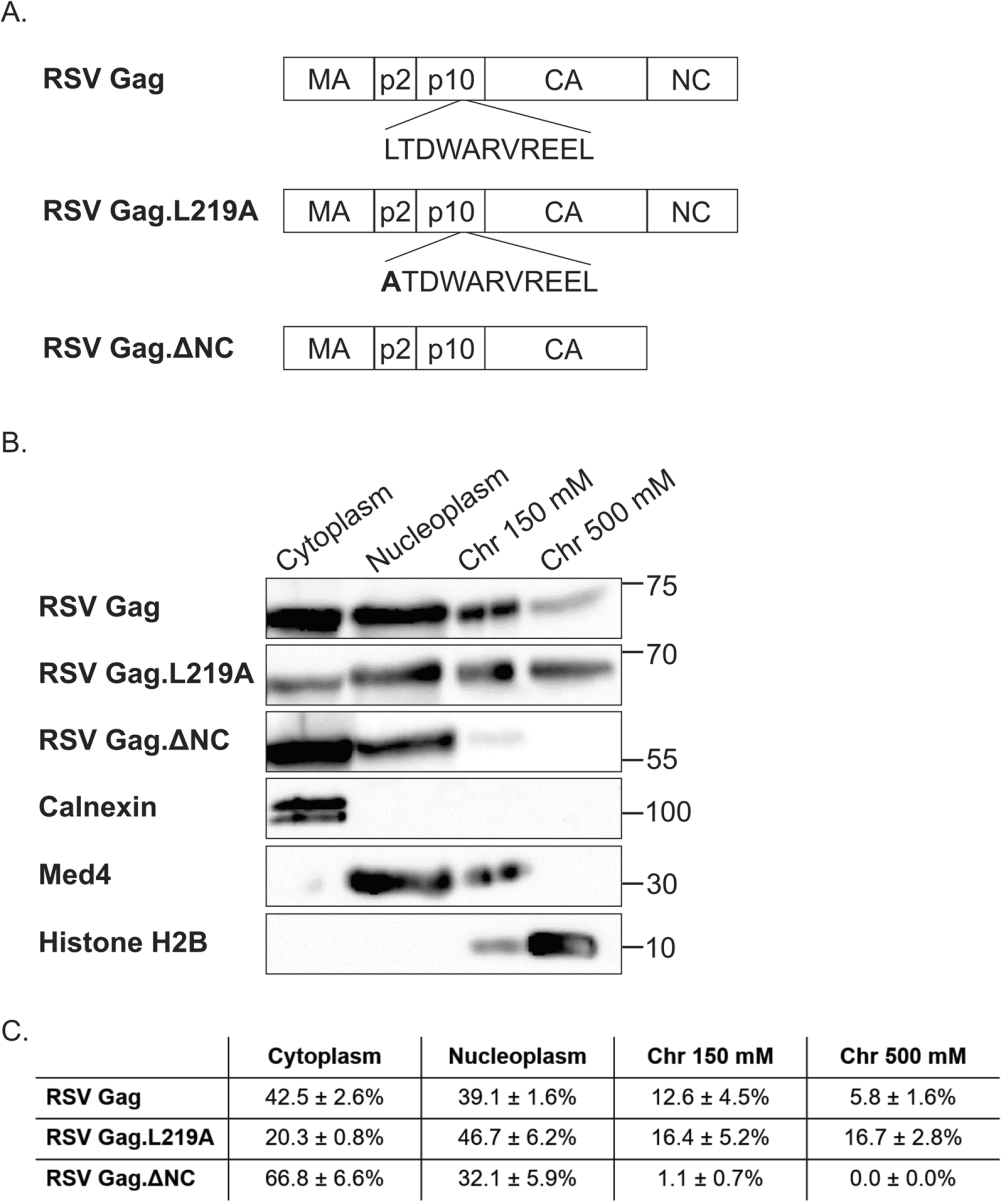
Schematic diagram and western blot with quantitation. **A** RSV Gag constructs. **B** Subcellular fractionations were performed to separate the cytoplasm and nucleoplasm from the chromatin fractions, which were further separated using differential NaCl concentrations. The 150 mM chromatin fraction (Chr 150 mM) contains proteins associated with open chromatin (euchromatin). The 500 mM chromatin fraction (Chr 500 mM) contains proteins that are associated with condensed chromatin (heterochromatin). Wild type RSV Gag and Gag.L219A were detected in all of the fractions at different ratios. Gag.ΔNC was primarily detected in the cytoplasm with very little in the chromatin fractions. **C** Band densities were determined for each Gag construct for each fraction and are displayed as mean ± standard error of the mean (SEM) for three biological replicates. To assess fraction purity, cellular proteins were detected using antibodies against calnexin (cytoplasm), Med4 (nucleoplasm and euchromatin), and Histone H2B (euchromatin and heterochromatin). The position of molecular weight markers, in kilodaltons, are indicated on the right

Prior experiments using microscopy revealed that both RSV and HIV-1 Gag proteins localize to the perichromatin compartment of the nucleus where they associate with their cognate USvRNAs at active transcription sites [3, 9, 11]. In addition, HIV-1 Gag associates preferentially with marks associated with transcriptionally-active euchromatin compared to heterochromatin [10]. To further define the nuclear subcompartments where RSV Gag is localized, cells expressing wild-type or mutant Gag proteins were separated into cytoplasmic, nucleoplasmic, and chromatin-associated protein fractions (Fig. 1). The first chromatin fraction was extracted using a NaCl concentration of 150 mM, which removes proteins that are loosely associated with chromatin (i.e., the euchromatin fraction). In the second chromatin fraction, a higher salt concentration was used (500 mM NaCl) along with detergent, to remove proteins that are more tightly bound to chromatin [52] (i.e., the heterochromatin fraction).

The signals of the protein bands were quantified to yield the relative ratio of Gag protein in each fraction, demonstrating that RSV Gag was present in the cytoplasm (42.5 ± 2.6%) and the nucleoplasm (39.1 ± 1.6%) of cells (Fig. 1A). Interestingly, RSV Gag was also present in both chromatin-associated protein fractions (euchromatin, 12.6 ± 4.5%; heterochromatin, 5.8 ± 1.6%). Examination of the nuclear-restricted mutant Gag.L219A, which contains a point mutation in the p10 NES, demonstrated decreased signal in the cytoplasm (20.3 ± 0.8%) and increased signal in both the nucleoplasm (46.7 ± 6.2%) and heterochromatin-associated fractions (16.7 ± 2.8%), compared to wild type Gag. Analysis of mutant Gag.ΔNC, which lacks the NC domain required for genomic RNA packaging, demonstrated an increase in cytoplasmic Gag compared to wild type (66.8 ± 6.6%), with little to no Gag.ΔNC in either of the chromatin fractions. Controls for the fractions included Calnexin (endoplasmic reticulum/cytoplasm), Mediator subunit 4 (Med4; nucleoplasm and euchromatin), and histone 2B (chromatin fractions). We have observed similar localization of HIV-1 Gag to all four subcellular compartments, as reported [9].

### Affinity purification and proteomic analysis

We next set out to identify potential nuclear interacting partners of RSV and HIV-1 Gag to provide clues about their possible role(s) in the nucleus. Recombinant His-tagged RSV and HIV-1 Gag proteins purified from *E. coli* were incubated with nuclear lysates from DF1 chicken fibroblast cells or HeLa cells, respectively. A beads-only control was also performed using DF1 lysates and HeLa lysates incubated with nickel beads. The affinity purifications were performed twice for both Gag proteins, as well as the beads-only controls. Proteins identified using mass spectrometry were combined into a single list for each Gag protein and analyzed using DAVID and Ingenuity Pathway Analysis, as described in Materials and Methods. Proteins identified from the beads-only purifications were removed from the lists of RSV and HIV-1 affinity-tagged purifications. A potential caveat and limitation to our experimental approach is that recombinant Gag proteins were added in excess to the nuclear factors in the lysates, which could lead to nonspecific interactions. To minimize this possibility and remove nonspecific proteins from the analysis, proteins that had an unused score of less than 1.3, as defined by ProteinPilot, were removed, along with common contaminants found in proteomics studies (see Supplemental files S1, S2, S3, and S4 for raw data).

DAVID was used to assign each protein from the two final Gag proteome lists to their cellular compartment(s) and biological process categories [16, 19]. We used the functional annotation tool to determine the most relevant gene ontology (GO) terms associated with the proteomics lists. The order of the GO terms that were enriched in the Gag proteome lists are dependent upon the p-values for each GO term. The smaller the p-value, the more that particular GO term was enriched. Table S1 shows the results of using the DAVID analysis software to analyze the list of proteins obtained from the RSV Gag affinity purification using DF1 nuclear lysates, and the top 10 biological processes’ GO terms are displayed. Only nuclear proteins, as determined by DAVID, were analyzed to determine the enriched biological functions, and Table S2 shows the top 10 results. Included in the results were generic GO terms such as: GO:0034641 ~ cellular nitrogen compound metabolic process, GO:1,901,360 ~ organic cyclic compound metabolic process, and GO:0006807 ~ nitrogen compound metabolic process. There were also more specific GO terms that were enriched, including GO:0010467 ~ gene expression and GO:0006396 ~ RNA processing.

Next, the potential binding partners of HIV-1 Gag isolated from HeLa nuclear lysates were examined. The terms GO:0006396 ~ RNA processing and GO:0010467 ~ gene expression were among the top 10 GO biological process terms identified after DAVID analysis (Table S3). When only nuclear proteins were examined, the top 10 GO terms again included GO:0010467 ~ gene expression and GO:0006396 ~ RNA processing (Table S4).

The proteins identified for each RSV and HIV-1 Gag affinity purification were compared, and we found there were 57 proteins that overlapped from 317 total proteins from the RSV list and 436 total proteins from the HIV-1 list (Fig S1). Table 1 contains the names and functions of the 57 proteins in common between the RSV and HIV-1 Gag. The functions of the 57 proteins are varied, encompassing cell cycle progression, RNA methylation, RNA processing and splicing, cytoskeletal regulation, mRNA nuclear export, transcription, DNA repair, and chromatin modification.

**Table 1:** Names and functions of the 57 cellular proteins identified in both the RSV and HIV-1 Gag protein interactomes

Symbol	Description	Function [summarized from Genecards [62] or NCBI]
ABLIM1	Actin Binding LIM Protein 1	A LIM zinc-binding domain-containing protein that binds to actin filaments. Mediates interactions between actin and cytoplasmic targets
ACTN1	Actinin Alpha 1	A bundling F-actin cross-linking protein thought to anchor actin to a variety of intracellular structures
ATXN2	Ataxin 2	Involved in EGFR trafficking, acting as negative regulator of endocytic EGFR internalization at the plasma membrane. modulates mTOR signals, modifying ribosomal translation and mitochondrial function
BCL7B	BAF Chromatin Remodeling Complex Subunit BCL7B	Positive regulator of apoptosis. Negatively regulates the expression of Wnt signaling components CTNNB1 and HMGA1. Involved in cell cycle progression, maintenance of the nuclear structure and stem cell differentiation
CBLL1	Cbl Proto-Oncogene Like 1	E3 ubiquitin-protein ligase that mediates ubiquitination of several tyrosine-phosphorylated Src substrates. Associated component of the WMM complex, a complex that mediates N6-methyladenosine (m6A) methylation of RNAs, a modification that has a role in the efficiency of mRNA splicing and RNA processing
CDC42BPB	CDC42 Binding Protein Kinase Beta	Serine/threonine-protein kinase which is an important downstream effector of CDC42 and plays a role in the regulation of cytoskeleton reorganization and cell migration
CHTOP	Chromatin Target of PRMT1	Plays an important role in the ligand-dependent activation of estrogen receptor target genes. Binds to 5-hydroxymethylcytosine (5hmC) and associates with the methylosome complex. The CHTOP-methylosome complex associated with 5hmC is recruited to selective sites on the chromosome, where it methylates H4R3 and activates the transcription of genes involved in glioblastomagenesis. Required for effective mRNA nuclear export and is a component of the TREX complex which is thought to couple mRNA transcription, processing and nuclear export, and specifically associates with spliced mRNA and not with unspliced pre-mRNA
CYFIP1	Cytoplasmic FMR1 Interacting Protein 1	Component of the CYFIP1-EIF4E-FMR1 complex which binds to the mRNA cap and mediates translational repression. Regulates formation of membrane ruffles and lamellipodia. Plays a role in axon outgrowth. Part of the WAVE complex that regulates actin filament reorganization via its interaction with the Arp2/3 complex
EIF4G2	Eukaryotic Translation Initiation Factor 4 Gamma 2	Plays a role in the switch from cap-dependent to IRES-mediated translation during mitosis, apoptosis and viral infection
FAM120A	Family with Sequence Similarity 120A	Critical component of the oxidative stress-induced survival signaling. Activates src family kinases and acts as a scaffolding protein enabling src family kinases to phosphorylate and activate PI3-kinase. Binds RNA and promotes the secretion of IGF-II
FOXP1	Forkhead Box P1	Essential transcriptional regulator of B-cell development. Involved in regulation of cardiac muscle cell proliferation. Involved in the columnar organization of spinal motor neurons. Represses transcription of various pro-apoptotic genes and cooperates with NF-kappa B-signaling in promoting B-cell expansion by inhibition of caspase-dependent apoptosis. Involved in endothelial cell proliferation, tube formation and migration indicative for a role in angiogenesis. Involved in transcriptional regulation in embryonic stem cells (ESCs). Stimulates expression of transcription factors that are required for pluripotency and decreases expression of differentiation-associated genes
HNRNPH3	Heterogeneous nuclear ribonucleoprotein H3	Involved in the splicing process and participates in early heat shock-induced splicing arrest
IGF2BP3	Insulin Like Growth Factor 2 MRNA Binding Protein 3	RNA-binding factor that may recruit target transcripts to cytoplasmic protein-RNA complexes (mRNPs) for mRNA transport and transient storage. It also modulates the rate and location at which target transcripts encounter the translational apparatus and shields them from endonuclease attacks or microRNA-mediated degradation
ING2	Inhibitor of Growth Family Member 2	Seems to be involved in p53/TP53 activation and p53/TP53-dependent apoptotic pathways, probably by enhancing acetylation of p53/TP53. Component of a mSin3A-like corepressor complex, which is probably involved in deacetylation of nucleosomal histones
IQGAP1	IQ Motif Containing GTPase Activating Protein 1	Plays a crucial role in regulating the dynamics and assembly of the actin cytoskeleton
KDM2A	Lysine Demethylase 2A	Histone demethylase that specifically demethylates 'Lys-36' of histone H3, thereby playing a central role in histone code. Required to maintain the heterochromatic state. Associates with centromeres and represses transcription of small non-coding RNAs that are encoded by the clusters of satellite repeats at the centromere. Required to sustain centromeric integrity and genomic stability, particularly during mitosis
KIAA1033 (WASHC4)	WASH Complex Subunit 4	Acts as a component of the WASH core complex that functions as a nucleation-promoting factor (NPF) at the surface of endosomes, where it recruits and activates the Arp2/3 complex to induce actin polymerization, playing a key role in the fission of tubules that serve as transport intermediates during endosome sorting
KLC1	Kinesin Light Chain 1	Kinesin is a microtubule-associated force-producing protein that may play a role in organelle transport. The light chain may function in coupling of cargo to the heavy chain or in the modulation of its ATPase activity
KLC2	Kinesin Light Chain 2	See above
LEO1	LEO1 Homolog, Paf1/RNA Polymerase II Complex Component	Component of the PAF1 complex (PAF1C) which has multiple functions during transcription by RNA polymerase II and is implicated in regulation of development and maintenance of embryonic stem cell pluripotency. PAF1C associates with RNA polymerase II through interaction with POLR2A CTD non-phosphorylated and 'Ser-2'- and 'Ser-5'- phosphorylated forms and is involved in transcriptional elongation
MAP2	Microtubule Associated Protein 2	The exact function of MAP2 is unknown but MAPs may stabilize the microtubules against depolymerization
MAPRE2	Microtubule Associated Protein RP/EB Family Member 2	May be involved in microtubule polymerization, and spindle function by stabilizing microtubules and anchoring them at centrosomes
MED26	Mediator Complex Subunit 26	Component of the Mediator complex, a coactivator involved in the regulated transcription of nearly all RNA polymerase II-dependent genes. Mediator functions as a bridge to convey information from gene-specific regulatory proteins to the basal RNA polymerase II transcription machinery
NDUFA9	NADH:Ubiquinone Oxidoreductase Subunit A9	Accessory subunit of the mitochondrial membrane respiratory chain NADH dehydrogenase (Complex I). Complex I functions in the transfer of electrons from NADH to the respiratory chain
OGT	O-Linked N-Acetylglucosamine (GlcNAc) Transferase	Catalyzes the transfer of a single N-acetylglucosamine from UDP-GlcNAc to a serine or threonine residue in cytoplasmic and nuclear proteins resulting in their modification with a beta-linked N-acetylglucosamine (O-GlcNAc). Glycosylates a large and diverse number of proteins and can regulate their cellular processes via cross-talk between glycosylation and phosphorylation or by affecting proteolytic processing. Component of a THAP1/THAP3-HCFC1-OGT complex that is required for the regulation of the transcriptional activity of RRM1. Plays a key role in chromatin structure by mediating O-GlcNAcylation of 'Ser-112' of histone H2B: recruited to CpG-rich transcription start sites of active genes via its interaction with TET proteins
ORC5	Origin Recognition Complex Subunit 5	Component of the origin recognition complex (ORC) that binds origins of replication. ORC is required to assemble the pre-replication complex necessary to initiate DNA replication
PDCD11	Programmed Cell Death 11	Essential for the generation of mature 18S rRNA. Directly interacts with U3 snoRNA. Involved in the biogenesis of rRNA
PDLIM5	PDZ And LIM Domain 5	May play an important role in the heart development by scaffolding PKC to the Z-disk region. May play a role in the regulation of cardiomyocyte expansion. Contributes to the regulation of dendritic spine morphogenesis in neurons
PLOD2	Procollagen-Lysine, 2-Oxoglutarate 5-Dioxygenase 2	Forms hydroxylysine residues in -Xaa-Lys-Gly- sequences in collagens. These hydroxylysines serve as sites of attachment for carbohydrate units and are essential for the stability of the intermolecular collagen cross-links
POLR2B	RNA Polymerase II Subunit B	DNA-dependent RNA polymerase catalyzes the transcription of DNA into RNA using the four ribonucleoside triphosphates as substrates. Second largest component of RNA polymerase II which synthesizes mRNA precursors and many functional non-coding RNAs. Proposed to contribute to the polymerase catalytic activity and forms the polymerase active center together with the largest subunit
PPIB	Peptidylprolyl Isomerase B	PPIase that catalyzes the cis–trans isomerization of proline imidic peptide bonds in oligopeptides and may therefore assist protein folding
PRPF40A	Pre-MRNA Processing Factor 40 Homolog A	Binds to WASL/N-WASP and suppresses its translocation from the nucleus to the cytoplasm, thereby inhibiting its cytoplasmic function. Plays a role in the regulation of cell morphology and cytoskeletal organization
PTBP1	Polypyrimidine Tract Binding Protein 1	Plays a role in pre-mRNA splicing and in the regulation of alternative splicing events. May promote RNA looping when bound to two separate polypyrimidine tracts in the same pre-mRNA. May promote the binding of U2 snRNP to pre-mRNA
RAD21	RAD21 Cohesin Complex Component	A member of the cohesin complex, involved in sister chromatid cohesion from the time of DNA replication in S phase to their segregation in mitosis, a function that is essential for proper chromosome segregation, post-replicative DNA repair, and the prevention of inappropriate recombination between repetitive regions
RBM33	RNA Binding Motif Protein 33	*Could not find a clear function associated with the protein
RFC1	Replication Factor C Subunit 1	The elongation of primed DNA templates by DNA polymerase delta and epsilon requires the action of the accessory proteins PCNA and activator 1. This subunit binds to the primer-template junction. Can bind single- or double-stranded DNA. 5' phosphate residue is required for binding of the N-terminal DNA-binding domain to duplex DNA, suggesting a role in recognition of non-primer template DNA structures during replication and/or repair
RFC5	Replication Factor C Subunit 5	See above
SAFB	Scaffold Attachment Factor B	Binds to scaffold/matrix attachment region (S/MAR) DNA and forms a molecular assembly point to allow the formation of a 'transcriptosomal' complex (consisting of SR proteins and RNA polymerase II) coupling transcription and RNA processing. Can function as an estrogen receptor corepressor and can also bind to the HSP27 promoter and decrease its transcription. Can inhibit cell proliferation
SAFB2	Scaffold Attachment Factor B2	See above with S/MAR DNA. Is involved in cell cycle regulation, apoptosis, differentiation, the stress response, and regulation of immune genes
SCAF8	SR-Related CTD Associated Factor 8	Anti-terminator protein required to prevent early mRNA termination during transcription. Mechanistically, associates with the phosphorylated C-terminal heptapeptide repeat domain (CTD) of the largest RNA polymerase II subunit (POLR2A), and subsequently binds nascent RNA upstream of early polyadenylation sites to prevent premature mRNA transcript cleavage and polyadenylation. Independently of SCAF4, also acts as a positive regulator of transcript elongation
SEPT7	Septin 7	Filament-forming cytoskeletal GTPase. Required for normal organization of the actin cytoskeleton. Required for normal progress through mitosis
SERBP1	SERPINE1 MRNA Binding Protein 1	May play a role in the regulation of mRNA stability. Seems to play a role in PML-nuclear bodies formation
SLC25A3	Solute Carrier Family 25 Member 3	Transport of phosphate groups from the cytosol to the mitochondrial matrix
SMAD2	SMAD Family Member 2	Receptor-regulated SMAD (R-SMAD) that is an intracellular signal transducer and transcriptional modulator activated by TGF-beta (transforming growth factor) and activin type 1 receptor kinases
SMC1A	Structural Maintenance of Chromosomes 1A	Involved in chromosome cohesion during cell cycle and in DNA repair. Involved in DNA repair via its interaction with BRCA1 and its related phosphorylation by ATM, or via its phosphorylation by ATR
SMC4	Structural Maintenance of Chromosomes 4	Central component of the condensin complex, a complex required for conversion of interphase chromatin into mitotic-like condense chromosomes
SURF4	Surfeit 4	May play a role in the maintenance of the architecture of the endoplasmic reticulum-Golgi intermediate compartment and of the Golgi
THOC3	THO Complex 3	Required for efficient export of polyadenylated RNA and spliced mRNA. Acts as component of the THO subcomplex of the TREX complex which is thought to couple mRNA transcription, processing and nuclear export, and which specifically associates with spliced mRNA and not with unspliced pre-mRNA
TJP1	Tight Junction Protein 1	TJP1, TJP2, and TJP3 are closely related scaffolding proteins that link tight junction (TJ) transmembrane proteins such as claudins, junctional adhesion molecules, and occludin to the actin cytoskeleton
TPM1	Tropomyosin 1	Binds to actin filaments in muscle and non-muscle cells. Plays a central role, in association with the troponin complex, in the calcium dependent regulation of vertebrate striated muscle contraction. In non-muscle cells is implicated in stabilizing cytoskeleton actin filaments
TPM3	Tropomyosin 3	Binds to actin filaments in muscle and non-muscle cells. Plays a central role, in association with the troponin complex, in the calcium dependent regulation of vertebrate striated muscle contraction. Smooth muscle contraction is regulated by interaction with caldesmon. In non-muscle cells is implicated in stabilizing cytoskeleton actin filaments
TRA2B	Transformer 2 Beta Homolog	Sequence-specific RNA-binding protein which participates in the control of pre-mRNA splicing. Can either activate or suppress exon inclusion. Binds to pre-mRNA
TRIP12	Thyroid Hormone Receptor Interactor 12	E3 ubiquitin-protein ligase involved in ubiquitin fusion degradation pathway and regulation of DNA repair. Acts as a key regulator of DNA damage
U2AF1	U2 Small Nuclear RNA Auxiliary Factor 1	Plays a critical role in both constitutive and enhancer-dependent splicing by mediating protein–protein interactions and protein-RNA interactions required for accurate 3'-splice site selection. Recruits U2 snRNP to the branch point. Directly mediates interactions between U2AF2 and proteins bound to the enhancers
VWA9 (INTS14)	Integrator Complex Subunit 14	Probable component of the Integrator (INT) complex, a complex involved in the small nuclear RNAs (snRNA) U1 and U2 transcription and in their 3'-box-dependent processing
ZEB1	Zinc Finger E-Box Binding Homeobox 1	Acts as a transcriptional repressor
ZFC3H1	Zinc Finger C3H1-Type Containing	Subunit of the trimeric poly(A) tail exosome targeting (PAXT) complex, a complex that directs a subset of long and polyadenylated poly(A) RNAs for exosomal degradation

### Comparative proteomic analysis of previously published HIV-1 Gag mass spectrometry experiments

Mass spectrometry experiments focusing on HIV-1 Gag cellular binding partners have been previously reported by other laboratories; however, these investigators did not focus on the nuclear proteins identified in these experiments. Engeland et al. [31] performed five independent affinity-tagged purification experiments to identify cellular proteins that interact with HIV-1 Gag. The techniques used consisted of a tandem affinity purification (TAP) tag for a C-terminally tagged Gag, GFP-TRAP A beads, and GFP microbeads for Gags with GFP fused either internally to the MA domain or the C-terminus of Gag. Each of these Gag constructs were transfected into 293 T cells and whole cell lysates were used for the affinity pulldowns. The authors found 31 proteins that were identified in at least 3 of the experiments, and of these 31 proteins, 24 localize to the nucleus. Using DAVID to analyze these 24 proteins, GO:0006396 ~ RNA processing and GO:0010467 ~ gene expression were the top two biological functions identified (Table S5). In 2014, Engeland and colleagues [30] examined the HIV-1 Gag interactome again and found 944 proteins that met their inclusion criteria. Using DAVID to analyze these 944 proteins, 186 were found to be nuclear. The authors performed a GO enrichment analysis using DAVID to determine the most enriched biological processes and found nuclear processes including GO:0006396 ~ RNA processing, GO:0008380 ~ RNA splicing, and GO:0006334 ~ nucleosome assembly. We performed a GO analysis using solely the 186 nuclear proteins identified and found similar results (Table S6).

In Jäger et al*.* [32], the authors identified host proteins that interact with each of the HIV-1 polyproteins, processed proteins, and accessory proteins, in a systematic and quantitative manner. A purification strategy was used that consisted of appending two Strep tags and three FLAG tags at the C-terminal end of each HIV-1 protein, which were expressed separately in HEK293 or Jurkat cells. When examining their raw data, we found that 1,134 unique proteins were identified from affinity purifications using full-length Gag plus the proteolytic cleavage products of Gag (MA, CA, NC, and p6). Of these interacting proteins, 180 were identified as being nuclear by DAVID analysis. Limiting our GO analysis using DAVID to these nuclear proteins, GO:0006396 ~ RNA processing was the top biological function term identified, followed by protein targeting functions and RNA metabolism (Table S7).

Ritchie et al. [35] provided further information on the possible protein interactors of HIV-1 Gag using the *E. coli* biotin ligase BirA* to permit identification of protein partners in close proximity to the bait protein [63–65]. The BirA* tag was inserted within the MA domain of Gag, and the Gag-BirA* construct was expressed in cells. The authors found 53 proteins after their exclusion criteria eliminated nonspecific interactors, and from these 53 proteins, we identified 17 nuclear proteins by DAVID analysis. Table S8 shows DAVID analysis of these 17 proteins, identifying the top biological categories of GO:0098609 ~ cell–cell adhesion, GO:0090304 ~ nucleic acid metabolic process, and GO:0010608 ~ posttranscriptional regulation of gene expression.

Le Sage et al. [33] also identified potential host factors that interact with HIV-1 Gag. Similar to Ritchie et al*.,* they used the BirA* tagging system, except the tag was placed at the N-terminus of Gag. They found a total of 42 proteins, of which 19 were nuclear. When these 19 nuclear proteins alone were analyzed by DAVID, the top hits included protein targeting and RNA processing and metabolism (Table S9).

Li et al. [34] examined binding partners of the MA domain of HIV-1 Gag by inserting a Strep tag at the C-terminus of MA and collecting MA-interacting complexes after HIV-1 infection. There were 97 proteins identified that met their inclusion criteria and were not present in the lysate-only control. Of these proteins, 63 were nuclear. When only the nuclear proteins were further analyzed by DAVID, the top categories were ER targeting and viral transcription/gene expression (Table S10).

There is one other publication that performed a tandem immunoprecipitation to identify HIV-1 Gag binding partners [66] that was not included in the comparative analysis presented here due to the small number of proteins identified. Gao et al*.,* identified 12 individual proteins and each of these were present in at least one of the previously discussed publications or found in the experiment we presented earlier in this report. They identified numerous ribosomal proteins, HNRNP proteins, and other nuclear proteins including histone proteins, EF1-α, nucleolin, B23, Nopp34, and SNRPD3.

To provide a comprehensive view of the set of proteins identified in multiple studies, proteins identified in the previously published laboratories’ reports were compared to the list of proteins found in our HIV-1 Gag affinity purifications. Table 2 shows the list of proteins identified in our HIV-1 Gag pulldown as well as at least one of the publications discussed above. Table 2 also indicates whether each protein was previously demonstrated to have a role in HIV-1 replication based on the HIV-1 human protein interaction dataset [67–69]. A total of 129 proteins identified in our mass spectrometry analysis were also present in at least one of the prior publications.

**Table 2:** Proteins identified in the present HIV-1 Gag affinity purification and found in at least one of the previously published HIV-1 Gag proteomic publications

Symbol	Description	Cell Compartment^+^	Involved in HIV replication^a^	No. of publications	*Ritchie *[35]	*Engeland 2014 *[30]	*Engeland 2011 *[31]	*Li *[34]	*Le Sage* [33]	*Jäger* [32]
MTDH	Metadherin	N/ER	Y	5	*	*	*		*	*
NAT10	N-acetyltransferase 10; RNA cytidine acetyltransferase	N	Y	4		*	*		*	*
ZC3HAV1	Zinc finger CCCH-type containing, antiviral 1	N/C	Y	4	*	*	*			*
ABCF1	ATP binding cassette subfamily F member 1	C/N	Y	3		*	*			*
CCT8	Chaperonin containing TCP1 subunit 8; T-complex protein 1 subunit theta	Ck/C	Y	3	*			*		*
CDC5L	Cell division cycle 5 like	C/N	Y	3		*	*			*
HNRNPM	Heterogeneous nuclear ribonucleoprotein M	N	Y	3	*	*				*
MAP4	Microtubule associated protein 4	Ck	Y	3	*	*				
MOV10	RISC complex RNA helicase	N/C	Y	3		*	*			*
NUFIP2	FMR1 interacting protein 2; Nuclear fragile X mental retardation-interacting protein 2	N/C	N	3	*	*				*
PLRG1	Pleiotropic regulator 1	N	N	3		*		*		*
PRPF3	Pre-mRNA processing factor 3; U4/U6 small nuclear ribonucleoprotein Prp3	N	N	3		*	*			*
PRPF4	Pre-mRNA processing factor 4; U4/U6 small nuclear ribonucleoprotein Prp4	N	N	3		*	*			*
RBM14	RNA binding motif protein 14	N/C	Y	3		*	*			*
RPL37A	60S ribosomal protein L37a	C/S/N	Y	3		*			*	*
SRPK1	SRSF protein kinase 1	N/C/ER	Y	3		*	*			*
SRSF3	Serine/arginine rich splicing factor 3	N/C	Y	3		*	*			*
SRSF7	Serine/arginine rich splicing factor 7	N/C	Y	3		*	*			*
TMPO	Thymopoietin; Lamina-associated polypeptide 2, isoform alpha	N	Y	3	*	*				*
TOP1	DNA topoisomerase I	N	Y	3		*	*			*
ALYREF	Aly/REF export factor; THO complex subunit 4	N/C	Y	2		*				*
ATAD3B	ATPase family, AAA domain containing 3B	M	Y	2		*				*
C7orf50	Chromosome 7 open reading frame 50	N	N	2		*				*
CHTOP	Chromatin target of PRMT1	N	N	2		*				*
DAP3	Death associated protein 3; 28S ribosomal protein S29, mitochondrial	M	Y	2		*				*
DDX24	DEAD-box helicase 24	N/Me	Y	2		*				*
DDX27	DEAD-box helicase 27	N	Y	2		*				*
DDX47	DEAD-box helicase 47	N	Y	2		*				*
DHX30	DExH-box helicase 30	M/C	Y	2		*				*
DIMT1	DIM1 dimethyladenosine transferase 1 homolog	N	Y	2		*				*
DKC1	Dyskerin pseudouridine synthase 1; H/ACA ribonucleoprotein complex subunit	N/C	Y	2		*				*
EIF2S1	Eukaryotic translation initiation factor 2 subunit alpha	C	Y	2		*				*
ELAVL1	ELAV like RNA binding protein 1	N/C	Y	2		*				*
EXOSC2	Exosome component 2	N/C	Y	2		*				*
FAM120A	Family with sequence similarity 120A; Constitutive coactivator of PPAR-gamma-like protein 1	PM/C	N	2		*				*
FBL	Fibrillarin; rRNA 2'-O-methyltransferase	N	N	2		*				*
FXR1	FMR1 autosomal homolog 1; Fragile X mental retardation syndrome-related protein 1	C	Y	2		*				*
G3BP2	G3BP stress granule assembly factor 2; Ras GTPase-activating protein-binding protein 2	C	Y	2		*				*
GRSF1	G-rich RNA sequence binding factor 1	M	Y	2		*				*
GRWD1	Glutamate rich WD repeat containing 1	N	N	2		*				*
GTF3C2	General transcription factor IIIC subunit 2	N	Y	2		*				*
H1FX	H1 histone family member X	N	Y	2		*				*
HIST1H1E	Histone cluster 1 H1 family member e	N	Y	2		*				*
HNRNPDL	Heterogeneous nuclear ribonucleoprotein D like	N/C	Y	2		*				*
HNRNPH3	Heterogeneous nuclear ribonucleoprotein H3	N	Y	2		*				*
HNRNPR	Heterogeneous nuclear ribonucleoprotein R	ER/N/C	Y	2		*				*
HNRNPUL1	Heterogeneous nuclear ribonucleoprotein U like 1	N	Y	2		*				*
IARS	Isoleucyl-tRNA synthetase	C	Y	2		*				*
IGF2BP1	Insulin like growth factor 2 mRNA binding protein 1	N/C	Y	2		*				*
IGF2BP3	Insulin like growth factor 2 mRNA binding protein 3	N/C	Y	2		*				*
ILF2	Interleukin enhancer binding factor 2	N/C	Y	2		*				*
ILF3	Interleukin enhancer binding factor 3	N/C	Y	2		*				*
LSM12	LSM12 homolog	C/N	Y	2		*				*
MKI67	Marker of proliferation Ki-67	N	Y	2	*					*
MRPS9	Mitochondrial ribosomal protein S9	M	Y	2		*				*
MRPS23	Mitochondrial ribosomal protein S23	M	Y	2		*				*
MRPS27	Mitochondrial ribosomal protein S27	M/C	N	2		*				*
MRPS31	Mitochondrial ribosomal protein S31	M	N	2		*				*
MRPS34	Mitochondrial ribosomal protein S34	M	N	2		*				*
NOP2	NOP2 nucleolar protein; Probable 28S rRNA (cytosine(4447)-C(5))-methyltransferase	N	N	2		*				*
NOP16	NOP16 nucleolar protein	N	N	2		*				*
PES1	Pescadillo ribosomal biogenesis factor 1	N	N	2		*				*
PDCD11	Programmed cell death 11	N	Y	2		*				*
PRRC2C	Proline rich coiled-coil 2C	C	Y	2		*	*			
PTCD3	Pentatricopeptide repeat domain 3	M	Y	2		*				*
PURA	Purine rich element binding protein A	N	Y	2		*				*
QARS	Glutaminyl-tRNA synthetase	C	Y	2		*				*
RBM10	RNA binding motif protein 10	N	Y	2		*				*
RFC1	Replication factor C subunit 1	N	Y	2		*				*
RFC5	Replication factor C subunit 5	N	Y	2		*				*
RPL5	60S ribosomal protein L5	C/N	Y	2		*				*
RPL15	60S ribosomal protein L15	Me	N	2		*				*
RPS24	40S ribosomal protein S24	C/ER/N/Me	Y	2		*				*
RPS28	40S ribosomal protein S28	C/ER	Y	2		*				*
RSL1D1	Ribosomal L1 domain containing 1	N	Y	2		*				*
SERBP1	SERPINE1 mRNA binding protein 1	N/C	N	2	*					*
SNRPA	U1 small nuclear ribonucleoprotein polypeptide A	N	Y	2		*				*
SPTAN1	Spectrin alpha, non-erythrocytic 1	Ck	Y	2		*				*
STRBP	Spermatid perinuclear RNA binding protein	C	Y	2		*				*
TRA2B	Transformer 2 beta homolog	N	Y	2		*				*
UPF1	RNA helicase and ATPase	N/C	Y	2		*				*
WDR3	WD repeat domain 3	N	Y	2		*				*
XRN1	5'–3' exoribonuclease 1	C	Y	2	*					*
ABLIM1	Actin binding LIM protein 1	Ck/C	N	1		*				
ACTN4	Actinin alpha 4	Ck/C/N	Y	1						*
AFG3L2	AFG3 like matrix AAA peptidase subunit 2	M	Y	1						*
AHNAK	Neuroblast differentiation-associated protein	N	Y	1	*					
ANKRD17	Ankyrin repeat domain 17	N/C	Y	1						*
ASUN (INTS13)	Asunder, spermatogenesis regulator; Integrator complex subunit 13	N/C	N	1		*				
BAG2	BCL2 associated athanogene 2	Ck/C	N	1						*
BAZ1A	Bromodomain adjacent to zinc finger domain 1A	N	Y	1						*
BAZ1B	Bromodomain adjacent to zinc finger domain 1B	N	Y	1						*
BSG	Basigin	PM/ER	Y	1						*
BUB3	Mitotic checkpoint protein	N	N	1						*
BYSL	Bystin like	N/C	N	1						*
C1QBP	Complement C1q binding protein	N/S/PM/M/C	Y	1						*
CCAR2	Cell cycle and apoptosis regulator 2	N/C	Y	1						*
CCDC86	Coiled-coil domain containing 86	N	N	1						*
CCT4	Chaperonin containing TCP1 subunit 4	Ck/C	Y	1						*
CCT5	Chaperonin containing TCP1 subunit 5	Ck/C	Y	1						*
CCT6A	Chaperonin containing TCP1 subunit 6A	C	Y	1						*
CCT7	Chaperonin containing TCP1 subunit 7	C	Y	1						*
CD3EAP	CD3e molecule associated protein; DNA-directed RNA polymerase I subunit RPA34	N	N	1						*
CDK12	Cyclin dependent kinase 12	N	N	1						*
CLTC	Clathrin heavy chain	Ck/C	Y	1						*
CORO1C	Coronin 1C	PM/Ck/Me	N	1						*
CTNNB1	Catenin beta 1	Ck/PM/N/C	Y	1		*				
DDX20	DEAD-box helicase 20	N/C	Y	1		*				
DDX31	DEAD-box helicase 31	N	N	1						*
DDX46	DEAD-box helicase 46	N/Me	Y	1						*
DNAJA3	DnaJ heat shock protein family (Hsp40) member A3	C/M/PM	N	1				*		
DNMT1	DNA methyltransferase 1	N	Y	1						*
ECPAS (KIAA0368)	Ecm29 Proteasome Adapter and Scaffold	ER/N/Ck	N	1		*				
EEF1B2	Eukaryotic translation elongation factor 1 beta 2	C	N	1						*
EXOSC4	Exosome component 4	C/N	N	1						*
FAU	Ubiquitin like and ribosomal protein S30 fusion	S/N/C	Y	1						*
FIP1L1	Factor interacting with PAPOLA and CPSF1	N	Y	1						*
GAPDH	Glyceraldehyde-3-phosphate dehydrogenase	C/Ck/N/Me	Y	1						*
GLYR1	Glyoxylate reductase 1 homolog	N	N	1						*
GPATCH4	G-patch domain containing 4	N/C	Y	1		*				
HIST1H1A	Histone cluster 1 H1 family member a	N	Y	1		*				
HIST2H2BF	Histone cluster 2 H2B family member f	N	Y	1		*				
HNRNPUL2	Heterogeneous nuclear ribonucleoprotein U like 2	N	N	1						*
HP1BP3	Heterochromatin protein 1 binding protein 3	N	N	1		*				
INIP	INTS3 and NABP interacting protein; SOSS complex subunit C	N	N	1		*				
INTS9	Integrator complex subunit 9	N	N	1				*		
ITPR1	Inositol 1,4,5-trisphosphate receptor type 1	ER/S	Y	1		*				
KIF4A	Chromosome-associated kinesin family member 4A	Ck/C/N	Y	1		*				
KIF23	Kinesin family member 23	N	Y	1						*
KLC2	Kinesin light chain 2	Ck/Me	N	1		*				
KRT2	Keratin 2	Ck/C/S/N/PM	Y	1						*
KRT16	Keratin 16	Ck/C/E/N	Y	1						*
KRT19	Keratin 19	Ck/C/E/PM	Y	1						*
LARP4	La ribonucleoprotein domain family member 4	C	Y	1						*
LARP7	La ribonucleoprotein domain family member 7	N	Y	1						*
MAPK1	Mitogen-activated protein kinase 1	Ck/C/N	Y	1						*
MBD2	Methyl-CpG binding domain protein 2	N	Y	1						*
MED26	Mediator complex subunit 26	N	Y	1		*				
MEPCE	7SK snRNA methylphosphate capping enzyme	N	Y	1						*
MINA (RIOX2)	MYC induced nuclear antigen; Ribosomal oxygenase 2	N	N	1						*
MRPL10	39S mitochondrial ribosomal protein L10	M	Y	1						*
MRPL53	Mitochondrial 39S ribosomal protein L53	M	Y	1						*
MRPS14	Mitochondrial 28S ribosomal protein S14	M	N	1						*
MRPS22	Mitochondrial 28S ribosomal protein S22	M	N	1		*				
NOC4L	Nucleolar complex associated 4 homolog	N	N	1						*
PABPN1	Poly(A) binding protein nuclear 1	N/C	Y	1						*
PAXBP1	PAX3 and PAX7 binding protein 1	N	Y	1						*
PBRM1	Polybromo 1	N	Y	1						*
PCBP1	Poly(rC) binding protein 1	C/N	Y	1						*
PPIH	Peptidylprolyl isomerase H	N/C	Y	1						*
PRRC2B	Proline rich coiled-coil 2B	N/C	N	1	*					
PSMC3	Proteasome 26S subunit, ATPase 3	N/C	Y	1		*				
PTBP1	Polypyrimidine tract binding protein 1	N	Y	1						*
PTRF (CAVIN1)	Polymerase I and transcript release factor; Caveolae-associated protein 1	C/N/ER/M/PM	N	1						*
PUM1	Pumilio RNA binding family member 1	C	N	1						*
RBM7	RNA binding motif protein 7	N	Y	1				*		
RBM26	RNA binding motif protein 26	N	Y	1	*					
RCC1	Regulator of chromosome condensation 1	N/C	Y	1		*				
RNF213	E3 ubiquitin-protein ligase; ring finger protein 213	C	Y	1		*				
RRP9	U3 small nucleolar RNA-interacting protein 2	N	Y	1						*
RSBN1L	Round spermatid basic protein 1 like; Lysine-specific demethylase	N	Y	1					*	
SDF2L1	Stromal cell derived factor 2 like 1	ER	Y	1						*
SEC24B	SEC24 homolog B, COPII coat complex component	C/ER	N	1						*
SLC25A3	Solute carrier family 25, member 3	M	Y	1						*
SLC25A4	Solute carrier family 25 member 4; ADP/ATP translocase 1	M/Me	Y	1						*
SLC25A11	Solute carrier family 25 member 11; Mitochondrial 2-oxoglutarate/malate carrier protein	M	Y	1						*
SMARCA4	SWI/SNF related, matrix associated, actin dependent regulator of chromatin; Transcription activator BRG1	N	Y	1						*
SMARCC2	SWI/SNF related, matrix associated, actin dependent regulator of chromatin subfamily c member 2	N	Y	1		*				
SMC4	Structural maintenance of chromosomes 4	C/N	Y	1		*				
SMN2	Survival of motor neuron 2, centromeric	N/C	N	1		*				
SNRPD2	Small nuclear ribonucleoprotein D2 polypeptide	C/N	Y	1						*
SPIN1	Spindlin 1	N	Y	1						*
SPOUT1	SPOUT domain containing methyltransferase 1	Ck	Y	1						*
SRP9	Signal recognition particle 9	C	Y	1						*
SUPT16H	SPT16 homolog, facilitates chromatin remodeling subunit	N	Y	1		*				
TAB1	TGF-beta activated kinase 1 (MAP3K7) binding protein 1	C/Me/N	N	1						*
TCEB1 (ELOC)	Transcription elongation factor B subunit 1; Elongin-C	N	Y	1						*
TCP1	t-complex 1	C/Ck	Y	1						*
TPM1	Tropomyosin 1 (alpha)	Ck	Y	1						*
TPM3	Tropomyosin 3	Ck	Y	1						*
TRIP12	Thyroid hormone receptor interactor 12; E3 ubiquitin-protein ligase	N	N	1						*
TRRAP	Transformation/transcription domain associated protein	N	Y	1		*				
TUBB6	Tubulin beta 6 class V	Ck	Y	1		*				
U2AF1	U2 small nuclear RNA auxiliary factor 1; Splicing factor U2AF 35 kDa	N	Y	1						*
UBAP2L	Ubiquitin associated protein 2 like	C	Y	1	*					
URB1 (NPA1)	Nucleolar pre-ribosomal-associated protein 1	N	Y	1						*
UTP20	UTP20, small subunit processome component	N	N	1						*
WDR5	WD repeat domain 5	N	Y	1						*
WDR82	WD repeat domain 82	N	Y	1		*				
ZC3H4	Zinc finger CCCH-type containing 4	C/N	N	1						*

+–C = cytoplasm, N: nucleus, M: mitochondria, PM: plasma membrane, Me: membrane, S: secreted, ER: endoplasmic reticulum, Ck: cytoskeleton; determined from UniProtKB (uniprot.org), or genecards.org [62] if localization not available on Uniprot.org; order determines abundance

^a^Summarized from HIV-1, human protein interaction dataset [65–67]

**Fig. 2:**
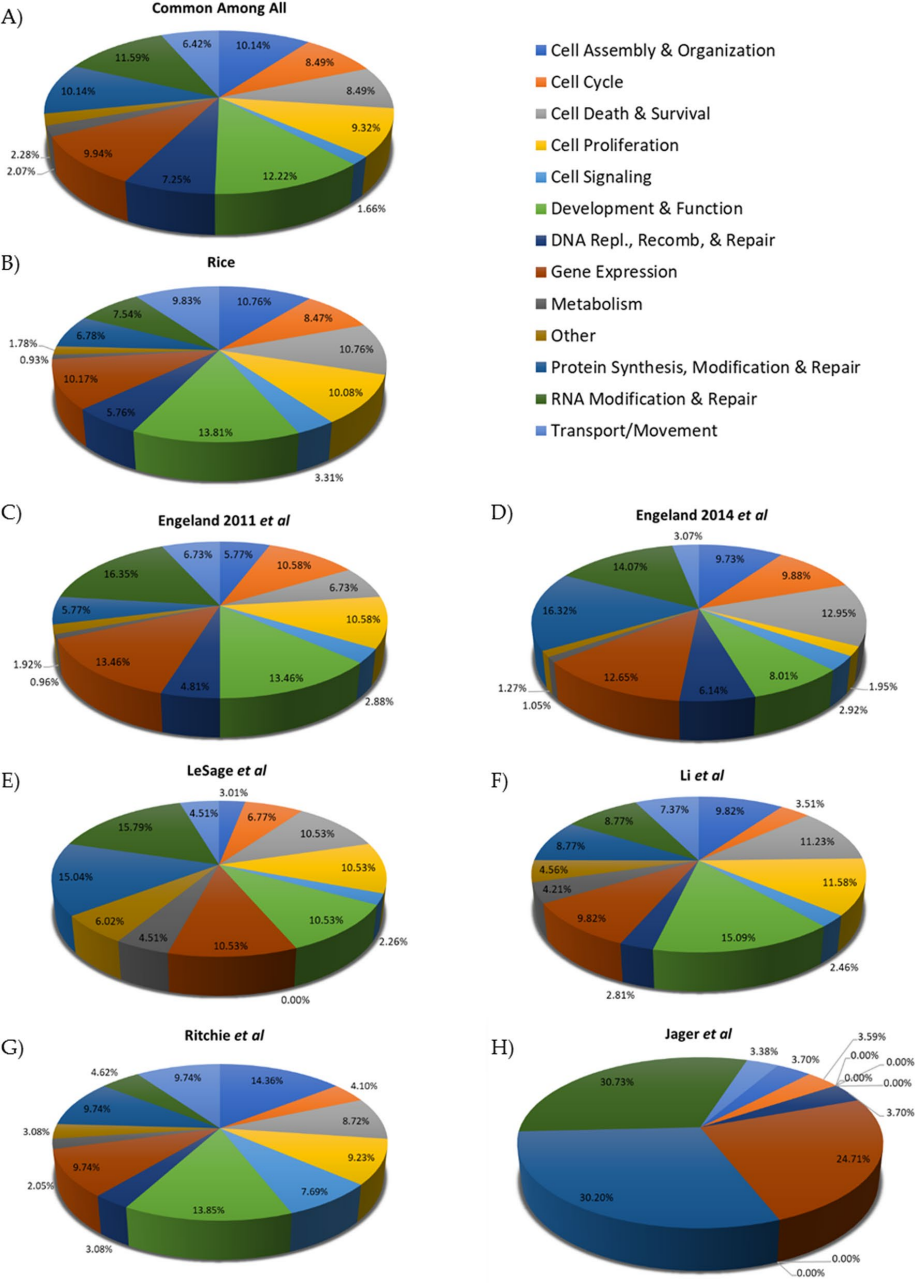
The relative representation of the IPA categories present in each protein list. **A** The proteins identified in our HIV-1 Gag pulldown that were also found in at least one of the previously published reports were analyzed for molecular and cellular functions. The color key on the right is the same for each pie graph. Protein functions identified in this publication (Rice) (**B**), Engeland 2011 [31] (**C**), Engeland 2014 [30] (**D**), Le Sage [33] (**E**), Li [34] (**F**), Ritchie [35] (**G**), and Jäger [32] (**H**)

These overlapping proteins were analyzed using Ingenuity Pathway Analysis (IPA) (Fig. 2), and the interacting proteins found in this report and at least one of the previously published lists were categorized based on their functions. Figure 2 shows the distribution of the proteins among 13 basic categories identified by Lippé [59]. Proteins may belong to more than one functional category, which results in the number of proteins present in each category exceeding the total number of proteins. To address this issue, the number of proteins in each category was divided by the total number of proteins to yield a percentage for each of the categories represented in the protein list analyzed. Figure 2 displays the relative values of the Ingenuity Pathway Analysis rather than the raw data.

### Closer examination of Gag interactions with host proteins involved in transcription and splicing

Numerous Gag-interacting proteins were found to be involved in RNA polymerase II transcription (GO term: 0006366 transcription from RNA polymerase II promoter) and splicing (GO term: 0008380 RNA splicing). We were interested in these two GO terms because our previous work demonstrated that RSV and HIV-1 Gag localize to the perichromatin space and associate with newly-transcribed USvRNA [8, 9], suggesting that they may interface with transcription and splicing processes. In addition, in Rice et al*.*, we demonstrated that RSV Gag.L219A colocalizes with the splicing factors SF2 and SC35, and has similar mobility and dynamics as proteins residing in splicing speckles [70]. Maldonado et al*.* subsequently showed that RSV Gag forms discrete nuclear foci and interacts with USvRNA at active transcription sites [8]. Taken together with the proteomic data presented here, it is reasonable to propose that Gag proteins interact with cellular proteins involved in transcription and co-transcriptional processes such as splicing and RNA processing, at or near transcription sites. Tables S11 and S12 list the proteins involved in transcription and splicing, respectively, for the RSV pulldowns. Similarly, Tuffy et al*.* and Chang et al*.* [9, 10] both demonstrated that HIV-1 Gag localizes to transcriptionally active regions in HeLa cells and T cells reactivated from latency. Given these findings, it is feasible that HIV-1 Gag interacts with host nuclear factors involved in transcription, RNA processing, and chromatin remodeling. Tables S13 and S14 list the proteins in these categories identified in our HIV-1 pulldowns and indicate whether each protein was identified in any of the other publications. Splicing factors CBLL1, HNRNPH3, TRA2B, PTN1, and U2AF1 were in the list of 57 interacting proteins common to RSV and HIV-1 Gag, suggesting that Gag could modulate splicing or compete with splicing factors co-transcriptionally to promote synthesis of unspliced viral RNA for translation and packaging. Of note, proteins involved in processes such as splicing are included in the DAVID category of transcription interactome [71, 72] because transcription and splicing have been shown to be linked [73–76].

### Validation of RSV Gag-Med26 Interaction

We were intrigued by the finding that our proteomic analyses of interactors with RSV and HIV-1 Gag proteins identified several components of the Mediator complex (RSV: Med6, Med13L, Med22, Med24, Med26, Med30; HIV-1: Med9, Med13, Med15, Med21, Med23, Med26, Med28) (Tables S1–S4, S11, S13), a coactivator involved in the regulated transcription of nearly all RNAPII genes [61, 77–80]. The presence of multiple Mediator proteins in our dataset raised the likelihood that Gag may interact with this multiprotein complex. Furthermore, Mediator proteins have been shown to be exploited by other viruses and endogenous retroelements [38–45]. Interestingly, Med26 and Med30 are both metazoan-specific Mediator proteins, implying that Gag may display selectivity for metazoan-specific Mediator complexes over those with protozoan orthologs. Med26 and Med30 both also have critical roles in transcription, with Med26 responsible for the recruitment of important elongation factors to sites of transcription, and Med30 providing stabilization of the intact Mediator core [81, 82].

**Fig. 3:**
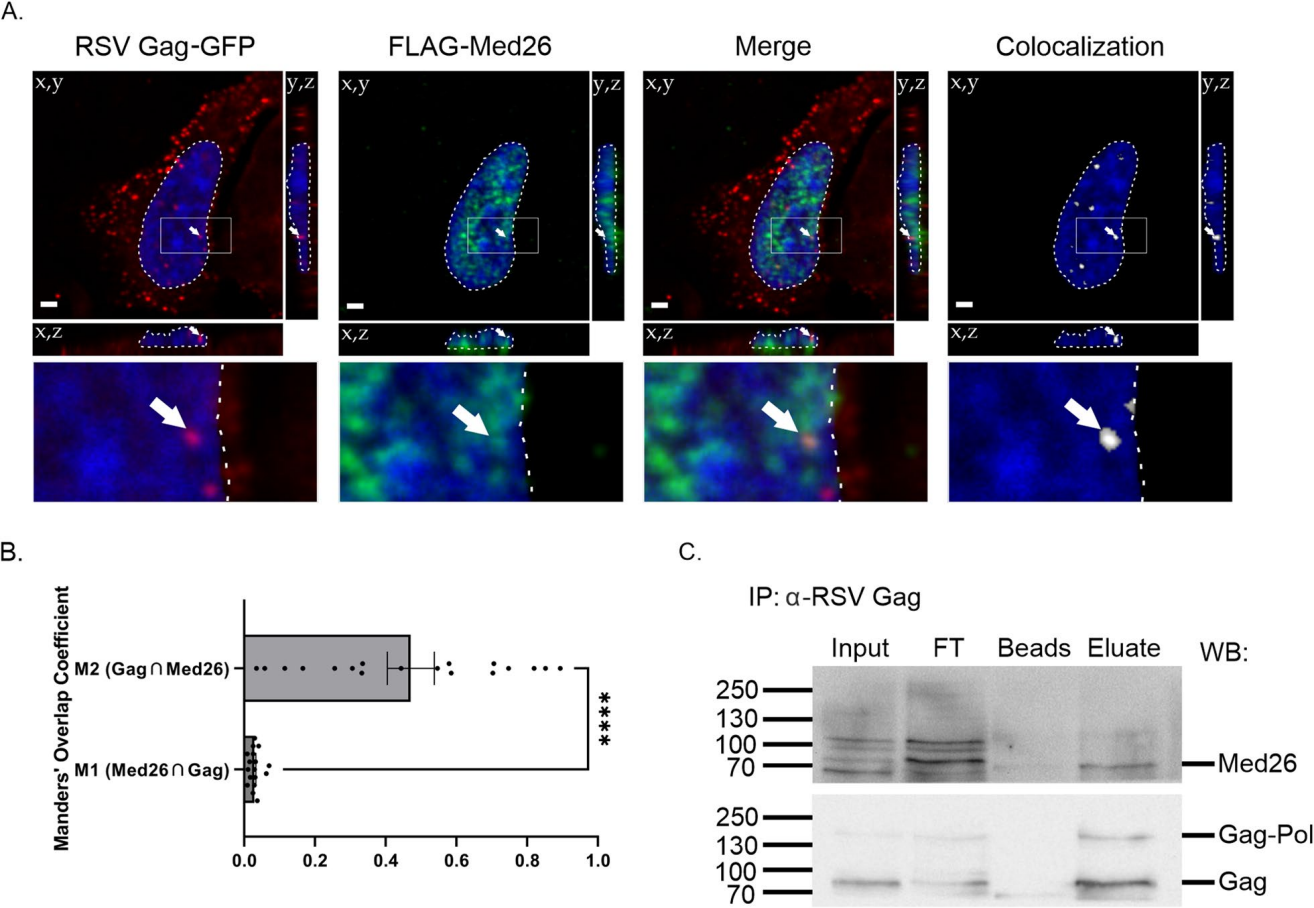
RSV Gag colocalized and co-immunoprecipitated with Med26. **A** Transfected RSV Gag-GFP (red) and FLAG-Med26 (green) colocalize (white) within QT6 cells. Image representative of average colocalization, as quantified in panel (**B**). Nuclei (blue) are outlined by a dotted white line, and regions boxed in the main images are enlarged below. White arrows are included to guide the eye. Scale bars = 2 µm. **B** Manders’ Overlap Coefficient values for image set represented by (**A**). Individual values are shown in addition mean ± SEM, n ≥ 17; ****, p < 0.0001 by unpaired two-tailed t-test. **C** 500 µg of RC.V8-infected QT6 nuclear lysates were incubated with an α-RSV Gag antibody (mouse α-RSV CA.A11, gift from Neil Christensen, Penn State College of Medicine), followed by antibody capture on Pierce™ Protein G Magnetic Beads. After extensive washing, proteins were eluted from beads by boiling in 1X SDS-PAGE sample buffer and run on a 10% SDS-PAGE gel, transferred to PVDF, and Western blotted first for Med26 (top) followed by RSV Gag (bottom). The position of molecular weight markers, in kilodaltons, are indicated on the left. FT, flow through; Beads, lysate only; Eluate, lysate plus antibody. Images representative of three independent experiments

We previously showed that nuclear localization of Gag contributes to efficient USvRNA selection for packaging [7]. One possible model is that interaction of Gag with Mediator proteins could tether Gag at active transcription sites, increasing the chance that it would find and associate with nascent USvRNA. To examine the interaction of RSV Gag and Med26, we utilized confocal microscopy to assess colocalization between transfected RSV Gag and Med26 in QT6 cells and observed that these two proteins did in fact colocalize (Fig. 3A, B, Video S1) (M1, Med26 ∩ RSV Gag: 0.0364 ± 0.004; M2, RSV Gag ∩ Med26: 0.471 ± 0.067). Next, immunoprecipitation of endogenous RSV Gag from RC.V8-infected QT6 cell nuclear lysates was undertaken, and samples were resolved via SDS-PAGE and subjected to western blotting, as described in the Methods section (Fig. 3C). In line with our proteomic results, we observed that Med26 and RSV Gag co-immunoprecipitated (Fig. 3C, Eluate lane). The molecular mechanisms underlying this interaction and the possible involvement of additional Mediator proteins will be pursued in future studies.

## Discussion

**Fig. 4:**
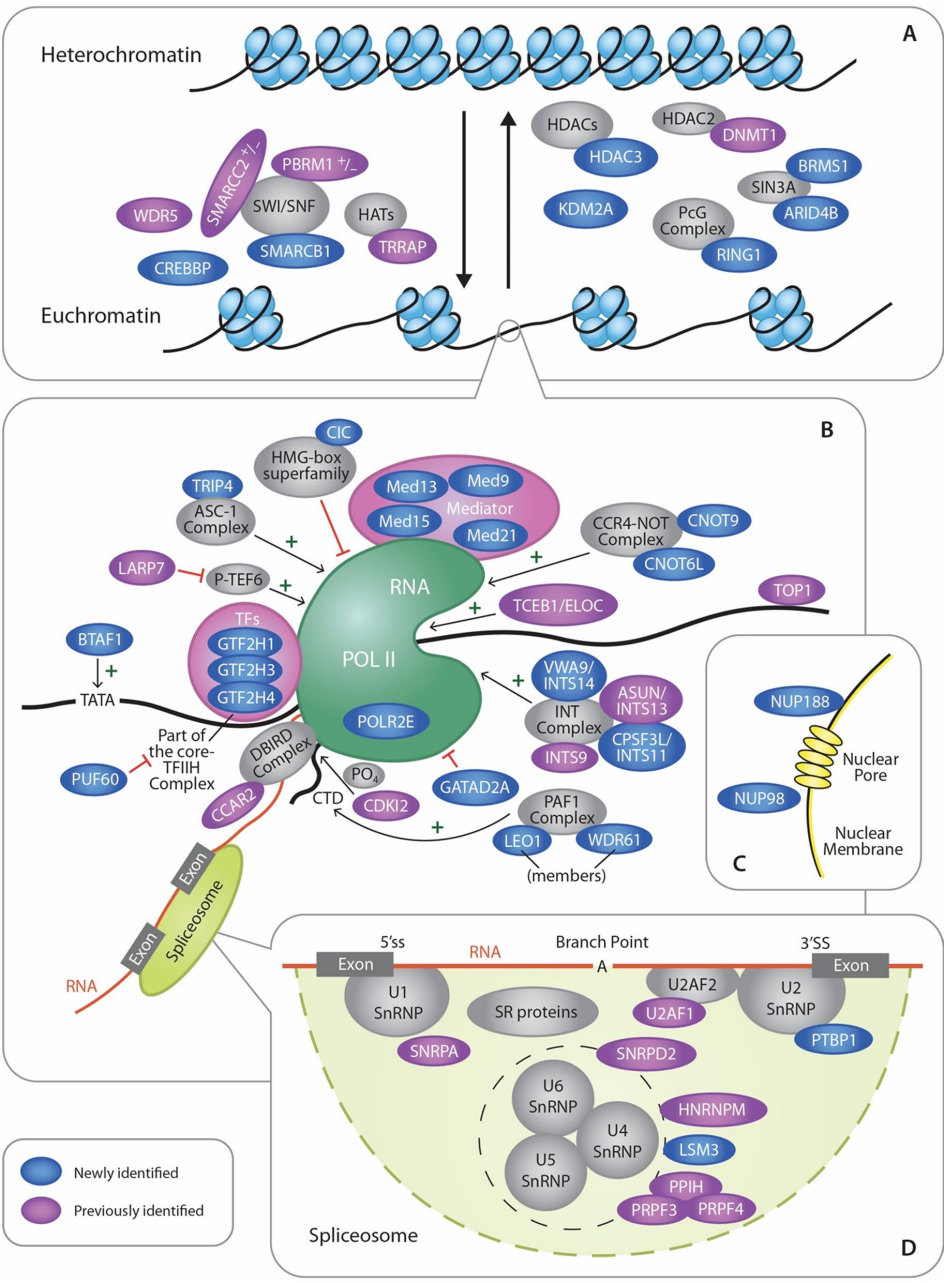
HIV-1 interactome pathway analysis. This diagram illustrates the HIV-1 Gag-interacting nuclear host factors discussed in this study. Factors shaded blue were uniquely identified in the present study (newly identified). Proteins shaded in purple were identified in this publication as well as at least one other published report (previously identified). Gray shading was used to show host protein complexes that are involved with Gag-interacting factors in chromatin remodeling, gene expression, nuclear export, and splicing. **A** HIV-1 Gag interacting factors that promote an open chromatin structure (euchromatin state) are on the left, whereas proteins involved in condensing chromatin are shown on the right. **B** Proteins involved in regulation of gene expression are depicted. Factors that promote transcription initiation are indicated by arrows and green plus signs. Factors that suppress or inhibit transcription are demarcated by red blocking lines. **C** The two nucleoporin proteins NUP98 and NUP188 were identified and are involved in trafficking between the nucleus and the cytoplasm through the nuclear pore complex. **D** Proteins that localize to the spliceosome and are involved in RNA splicing are shown

Several different laboratories have observed that the full-length Gag proteins of HIV-1, RSV, MMTV, MLV, FIV, PFV, and MPMV undergo nuclear localization [9–27, 83, 84]. As described in detail in this report, six previously published proteomic studies searching for binding partners of HIV-1 Gag identified many nuclear proteins [30–35]. As we were specifically interested in the nuclear interactomes of RSV and HIV-1 Gag, we took a different approach than previous groups, using nuclear lysates incubated with recombinant Gag proteins to perform affinity purification of complexes followed by mass spectrometry for our proteomic analysis. Despite these differences in methodology, a set of overlapping factors were identified for HIV-1 Gag that included a large number of proteins involved in nuclear processes such as transcription/gene expression, RNA processing, splicing, and chromatin remodeling. Comparison of the potential binding partners of RSV and HIV-1 Gag indicated that 57 proteins were found to be in common (Figure S1). Whether these factors have similar functions in RSV or HIV-1 replication remains to be examined. When the HIV-1 Gag interactomes identified by other laboratories were compared to the interacting proteins identified by us in this report, 190 common proteins were found by at least two independent laboratory groups. Further experimentation will be needed to validate each of these factors to determine whether they play important roles in retrovirus replication or pathogenesis (Fig. 4).

**Fig. 5:**
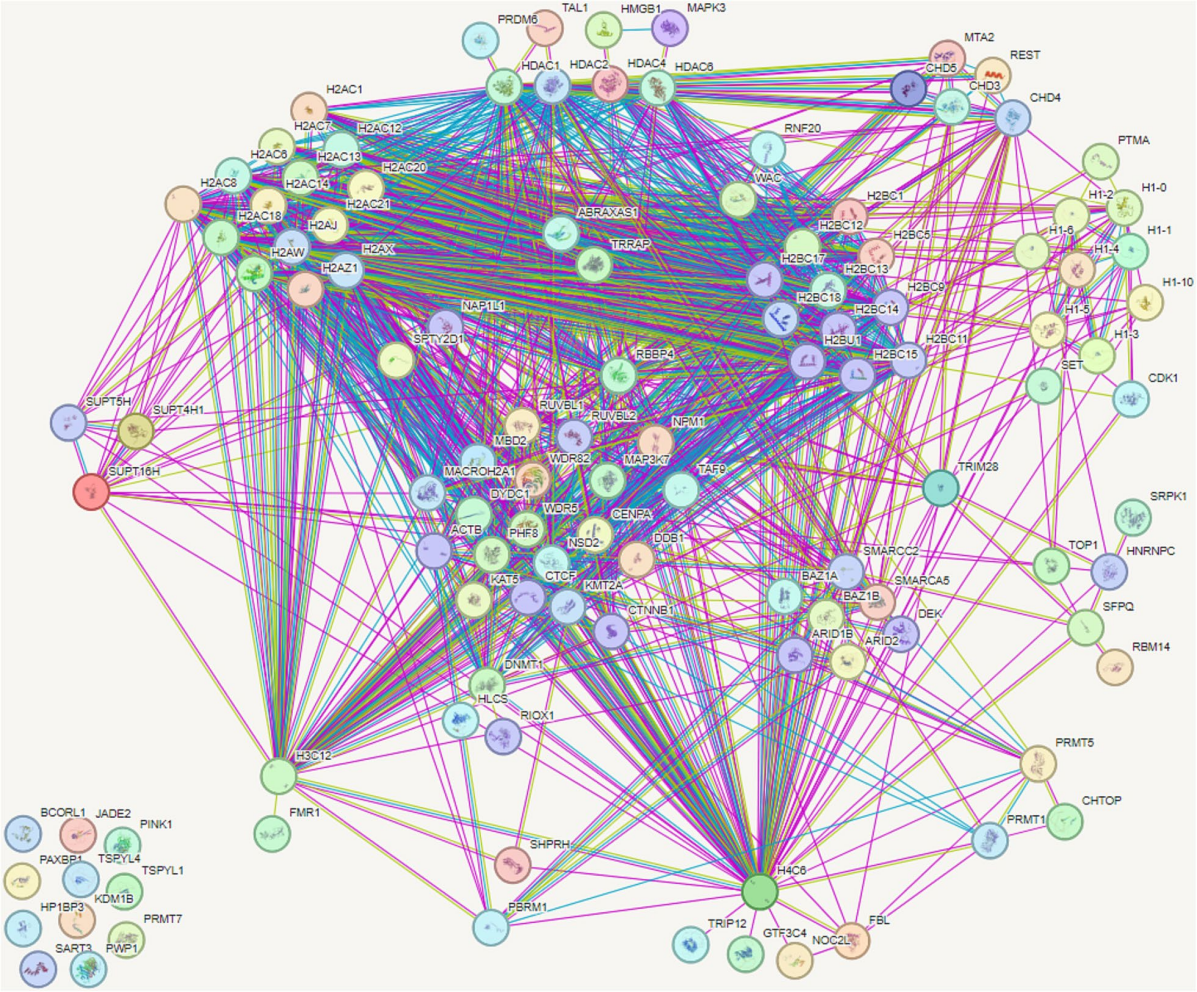
STRING protein network map of HIV-1 Gag interacting host chromatin proteins. Protein lists were generated from mass spectrometry experiments of Gag interacting proteins. Gene lists were then categorized into gene ontology (GO) terms to identify those which were chromatin associated. This refined list was input into STRING Consortium v12.0 (https://string-db.org/) to generate a protein–protein interaction map. A total of 129 proteins were queried and the following physical protein–protein interactions were made. Among the 129 proteins queried, 117 proteins are displayed after a minimum required interaction score of 0.4 was applied. Proteins that do not have any interacting partners are shown at the bottom left corner of the map. Lines are generated based on known interactions (purple lines), predicted gene fusions (red lines), predicted gene neighborhoods (green lines), and predicted gene co-occurrences (blue lines) from the literature

Our past and present cell fractionation experiments demonstrated that, in addition to being present in the nucleoplasm, both the RSV and HIV-1 Gag proteins can be extracted from euchromatin and heterochromatin fractions, complementing our recently published report indicating that HIV-1 Gag localizes with euchromatin marks at the nuclear periphery [9, 10]. These results suggest that HIV-1 Gag may be specifically targeted to a chromatin-associated compartment through interactions with host nuclear binding partners. Indeed, many of the proteins identified by us and others are chromatin-associated, raising the importance of further exploring the signals that target chromatin-associated regions, examining Gag interactions with chromatin factors, and elucidating possible roles for Gag in chromatin-related functions. To better understand how these chromatin-associated factors interact, and to identify which histone-associated protein networks appear most closely associated with HIV-1 Gag, we employed the use of the STRING Consortium database (Fig. 5). Of those identified, histones H1 (8 proteins), H2A (14 proteins), and H2B (11 proteins) displayed the strongest evidence for association. Histones H3 and H4 were also identified, but each only displayed a single association. Additionally, four histone deacetylases (HDACs)—including the transcription-repressing class II HDACs 4 and 6—and seven members of the SWI/SNF chromatin remodeling complex (e.g. SMARCC2, SMARCA5, DEK, BAZ1A, MAZ1B, ARID1B, and ARID2) were identified [85]. SWI/SNF complexes are highly enriched at transcriptional enhancer regions, where they modulate accessibility to promote gene activations [86]. Interestingly, SWI/SNF complexes have been shown to play a role in HIV-1 infection [87–89], and also interact with histones H2A, H2B, and H4. Given the spatial organization of the nucleosome, it is possible that HIV-1 Gag may be interacting with factors residing near histones H1, H2A, H2B, and H4, to locate viral transcription sites, influence transcription of viral or cellular genes, or facilitate chromatin remodeling to expose the integrated provirus.

It will be important to investigate whether these interactions facilitate viral replication steps occurring in the nucleus. Based on the proteins identified in the proteomic screens, we hypothesize that Gag proteins could modulate cellular functions including gene expression, viral RNA processing, splicing, or nuclear export of viral RNA complexes. Additionally, Gag might influence cellular processes for the benefit of the virus, for example by modulating expression of cellular genes involved in the immune response or by altering splicing patterns of host genes to change their localization or function. Gag proteins were not previously known to be involved in modulating transcription, splicing, RNA transport, or immune responses. Therefore regardless of which function(s) were found to be involved, these findings would be a significant step forward in our understanding of retroviral biology and provide additional targets for therapeutic intervention. Figure 4 highlights the various nuclear processes that HIV-1 Gag could be involved in based on the proteins identified in the HIV-1 proteomic studies.

Our published data indicating that RSV and HIV-1 Gag each interact specifically with their cognate USvRNAs to form discrete foci to form viral RNP complexes in the interchromatin space raise the possibility that interaction with host factors facilitates co-transcriptional retroviral genome selection [8, 9]. In both viruses, these findings were confirmed in infected cells, where Gag was targeted to the USvRNA transcription site, presumably at the site of proviral integration. Among other transcription-related proteins, in this study we identified multiple members of the Mediator complex, which has been shown to be hijacked by other viruses and retroelements, and we demonstrated that RSV Gag colocalized and co-immunoprecipitated with Med26 (Fig. 3 and Video S1) [38–45]. Quantitative analysis of our imaging experiments indicated that nearly half of the nuclear Gag signal was colocalized with Med26. Visual inspection of the colocalization channel revealed that much of this colocalization was at Gag foci in the perichromatin space (Fig. 3A). Our previous work demonstrating that RSV and HIV-1 Gag proteins can form biomolecular condensates (BMCs) [90, 91], combined with evidence for the existence of Mediator-containing transcriptional condensates [92, 93], suggests that Gag may use condensate-driven compartmentalization with Mediators and other transcription proteins to localize to sites of nascent viral RNA synthesis. Future studies to explore this possibility and to examine the biological function of transcription-related factors in Gag-mediated processes will be crucial for making important breakthroughs in understanding the role of the nuclear population of Gag proteins in virus replication.

Interestingly, we identified two components of the WMM complex, CBLL1 (Cbl Proto-Oncogene Like 1; present in RSV and HIV-1 datasets) and WTAP (Wilms tumor 1 associated protein; RSV dataset only), which mediates m6-methyladenosine (m6A) methylation of RNAs. This RNA modification affects RNA splicing and processing, and full length HIV-1 RNA containing m6A has displayed a bias towards serving as template RNA for the translation of viral proteins, as opposed to being packaged as gRNA [84]. Interaction with components of the WMM complex could simply serve to localize Gag to sites where viral RNA may exist, but it is intriguing to consider the possibility that Gag antagonizes the function of this complex to maintain a pool of genomic RNA.

For RSV, we have observed movement of viral RNPs across the nuclear membrane [8], suggesting that these complexes may ultimately be packaged into assembling virions at the plasma membrane, although it is also possible that these interactions with nuclear proteins are transient. We also previously found that RSV Gag colocalizes with splicing factors SF2 and SC35 [70] in splicing speckles, which are located in the perichromatin space, raising the possibility that Gag may influence splicing of viral and/or cellular RNAs in a co-transcriptional manner. It would be of interest to test the hypothesis that RSV Gag suppresses splicing at viral intron/exon junctions to promote retention of full-length viral RNA for use as the viral genome.

Tables S11–14 list the proteins involved in transcription and splicing identified in the RSV and HIV-1 Gag interactomes. We noted that there were Gag-interacting proteins identified at different stages of transcription, including initiation, elongation, and termination. Interestingly, PolR2B, the second largest subunit of RNA polymerase II (RNAPII), was identified in both the RSV and HIV-1 interactome datasets, raising the possibility that Gag interacts with RNAPII itself [94, 95]. Each dataset also contained a member of the Elongin complex (TCEB1, or Elongin C, for HIV-1; TCEB3, or Elongin A, for RSV), which forms a complex with PolR2B to promote elongation [96]. Numerous members of the Integrator complex were also identified in the datasets (VWA9, RSV and HIV-1; INTS3 and INTS5, RSV only; ASUN, INTS5, INTS9, and CPSF3L, HIV-1 only). Integrator regulates transcription by binding to the C-terminal tail of PolR2A (the largest subunit of RNAPII) during pausing, which results in cleavage of newly synthesized RNA and termination of transcription [97–99]. Other factors that modulate transcription identified were LEO1 and SCAF8 (both RSV and HIV-1), CDK13 and ZNF326 (RSV only), and ALYREF, CCAR2, CDK12, SETD2, and SUPT16H (HIV-1 only). One intriguing idea is that Gag could alter transcription elongation rates or regulate pausing of nascent viral RNA synthesis to promote folding of the psi region, recruit essential host RNA binding factors, modulate or suppress termination or splicing of viral RNA, or alter other co-transcriptional processes that could have downstream effects on the fate of viral RNA.

Although RSV and HIV-1 Gag are found in the nucleus, the mechanism of transport remains unknown. We identified KLC1 and KLC2 (AlphaFold Protein Structure Database [36, 37], entries Q07866/Q9H0B6) in the list of 57 proteins in common to both Gag proteins, suggesting that kinesin motor proteins could mediate nuclear entry, intranuclear transport, and/or nuclear export. Of interest, nuclear entry and uncoating of the HIV-1 preintegration complex are mediated by the kinesin motor proteins (reviewed in [100]), suggesting this class of proteins may be important for nuclear entry of both retroviral capsids and Gag proteins. Additionally, several studies have demonstrated that the intact HIV-1 capsid core enters the nucleus [101–103] and interacts with CPSF6 and Nup153 to transport the preintegration complex to the site of integration [104–108]. Two recent reports that may have relevance for full-length Gag nuclear trafficking show that the mature HIV -1 CA protein, when assembled into hexamers, serves as its own nuclear transporter by functionally acting like karyopherins and interacting with phenylalanine-glycine (FG) repeats within a variety of nucleoporins (Nups) within the nuclear pore complex [109, 110]. Full length HIV-1 Gag contains the CA sequence, however its conformation in Gag appears to be different from that of the mature CA sequence after cleavage during maturation [111–114]. Therefore, although it is feasible that the immature Gag protein could interact with Nups in an indiscriminate manner like the CA core does, it is also possible that the immature Gag protein utilizes a different set of Nups to facilitate nuclear entry and egress. It is intriguing to speculate that HIV-1 Gag, which forms biomolecular condensates (BMCs) [91, 115], could move through nuclear pores by interacting with the FG repeats in Nups, which undergo phase separation and form BMCs [116]. As our pulldowns identified Nup98 and Nup188 as possible interactors with HIV-1 Gag, it is possible that the nuclear trafficking of HIV-1 Gag may involve BMC-related interactions, and further studies will need to be performed to address this intriguing possibility. For RSV Gag, we have shown that Nup98 and Nup214 are functionally important for nuclear export, although a direct interaction has not been shown [4] and will require further studies.

The data presented here, along with our analysis of previously published HIV-1 Gag interactomes, indicate that many potential host protein partners of RSV and HIV-1 Gag reside in the nucleus in association with chromatin. These findings represent a promising new avenue of investigation for the retroviral research community with exciting potential for improving patient outcomes if new therapies targeting nuclear processes were developed. As both RSV and HIV-1 Gag have nuclear populations that form vRNPs in the perichromatin region, the interaction of Gag proteins with chromatin-associated nuclear factors merits further investigation. Significant novel roles for Gag proteins of RSV, HIV-1, and other retroviruses may be uncovered, shedding light on previously unknown aspects of the replication cycle that could be targeted by antiviral therapies.

### Supplementary Information

Supplementary Material 1. RSV Gag Dataset 1.xlsx.

Supplementary Material 2. RSV Gag Dataset 2.xlsx.

Supplementary Material 3. HIV Gag Dataset 1.xlsx.

Supplementary Material 4. HIV Gag Dataset 2.xlsx.

Supplementary Material 5: Table S1. Top 10 DAVID biological processes for proteins isolated from the RSV Gag affinity purifications from DF1 nuclear lysates.

Supplementary Material 6: Table S2. Top 10 nuclear enriched DAVID biological processes for proteins isolated from the RSV Gag affinity purifications from DF1 nuclear lysates.

Supplementary Material 7: Table S3. Top 10 DAVID biological processes for proteins isolated from the purified HIV Gag affinity purifications from HeLa nuclear lysates.

Supplementary Material 8: Table S4. Top 10 nuclear enriched DAVID biological processes for proteins isolated from the purified HIV Gag affinity purifications from HeLa nuclear lysates

Supplementary Material 9: Table S5. Top 10 DAVID biological processes of nuclear proteins identified in Engeland et al. [31].

Supplementary Material 10: Table S6. Top 10 DAVID biological processes of nuclear proteins identified in Engeland et al. [30].

Supplementary Material 11: Table S7. Top 10 DAVID biological processes of nuclear proteins identified in Jäger et al. [32].

Supplementary Material 12: Table S8. Top 10 DAVID biological processes of nuclear proteins identified in Ritchie et al. [35].

Supplementary Material 13: Table S9. Top 10 DAVID biological processes of nuclear proteins identified in Le Sage et al. [33].

Supplementary Material 14: Table S10. Top 10 DAVID biological processes of nuclear proteins identified in Li et al. [34].

Supplementary Material 15: Table S11. Names and functions of the proteins identified in the RSV proteomics list under GO:0006366 ~ transcription from RNA polymerase II promoter.

Supplementary Material 16: Table S12. Names and functions of the proteins identified in the RSV proteomics list under GO:0008380 ~ RNA splicing.

Supplementary Material 17: Table S13. Names and functions of the proteins identified in the HIV-1 proteomics list under GO:0006366 ~ transcription from RNA polymerase II promoter.

Supplementary Material 18: Table S14. Names and function of the proteins identified in the HIV-1 proteomics list under the GO:0008380 ~ RNA splicing.

Supplementary Material 19: Figure S1. The number of proteins identified in both the RSV and HIV-1 Gag affinity tagged purifications.

Supplementary Material 20: Video S1. RSV Gag and Mediator complex subunit 26 (Med26) colocalize in transfected QT6 cells.

## Data Availability

This information is available under the subheading “Protein identification and analysis”.
