# Supplementary material for: Comparative analysis of retroviral Gag-host cell interactions: focus on the nuclear interactome
Source: Retrovirology. 2024 Jun 19;21:13. doi: 10.1186/s12977-024-00645-y (PMC11186191; doi:10.1186/s12977-024-00645-y)
Supplement: Supplementary file 11 — Supplementary Material 11: Table S7. Top 10 DAVID biological processes of nuclear proteins identified in Jäger et al. [32]. [file 12977_2024_645_MOESM11_ESM.docx]

**Table S12.** Names and functions of the proteins identified in the RSV proteomics list under GO:0008380~RNA splicing.

| **Symbol** | **Description** | **Function related to Splicing [mostly summarized from Genecards (62)]** |
| --- | --- | --- |
| **AQR** | Aquarius intron-binding spliceosomal factor | Intron-binding spliceosomal protein required to link pre-mRNA splicing and snoRNP (small nucleolar ribonucleoprotein) biogenesis |
| **CACTIN** | Cactin, Spliceosome C Complex Subunit | Essential for efficient splicing of many precursor mRNAs (pre-mRNAs). Interacts with the spliceosome-associated factors DHX8 and SRRM2 (83). |
| **CDK13** | Cyclin Dependent Kinase 13 | Required for RNA splicing, probably by phosphorylating SRSF1/SF2. |
| **EIF4A3** | Eukaryotic Translation Initiation Factor 4A3 | Involved in pre-mRNA splicing as component of the spliceosome. Core component of the splicing-dependent multiprotein exon junction complex (EJC) deposited at splice junctions on mRNAs. |
| **FUS** | FUS RNA Binding Protein | Binds to nascent pre-mRNAs and acts as a molecular mediator between RNA polymerase II and U1 small nuclear ribonucleoprotein thereby coupling transcription and splicing. |
| **HNRNPH1** | Heterogeneous Nuclear Ribonucleoprotein H1 | Mediates pre-mRNA alternative splicing regulation. |
| **HNRNPH3** | heterogeneous nuclear ribonucleoprotein H3 | Involved in the splicing process and participates in early heat shock-induced splicing arrest. |
| **HNRNPK** | Heterogeneous Nuclear Ribonucleoprotein K | Member of the heterogeneous nuclear ribonucleoproteins (hnRNPs) family. The hnRNPs are RNA binding proteins and they complex with heterogeneous nuclear RNA (hnRNA). These proteins are associated with pre-mRNAs in the nucleus and appear to influence pre-mRNA processing and other aspects of mRNA metabolism and transport. |
| **ISY1** | ISY1 Splicing Factor Homolog | Component of the spliceosome C complex required for the selective processing of microRNAs during embryonic stem cell differentiation. |
| **LUC7L3** | LUC7-like 3 pre-mRNA splicing factor; human homolog of yeast U1 snRNP-associated factor | Acts as a bridge between the pre-mRNA and the U1 snRNP during splicing (84). |
| **MAGOH** | Mago Homolog, Exon Junction Complex Subunit | Required for pre-mRNA splicing as component of the spliceosome. Plays a redundant role with MAGOHB as core component of the exon junction complex (EJC) and in the nonsense-mediated decay (NMD) pathway. |
| **NONO** | Non-POU Domain Containing Octamer Binding | Involved in pre-mRNA splicing, probably as a heterodimer with SFPQ. Interacts with U5 snRNA. |
| **POLR2B** | RNA Polymerase II Subunit B | Second largest component of RNA polymerase II. |
| **PRPF8** | pre-mRNA processing factor 8 | A core component of precatalytic, catalytic and postcatalytic spliceosomal complexes, both of the predominant U2-type spliceosome and the minor U12-type spliceosome. Functions as a scaffold that mediates the ordered assembly of spliceosomal proteins and snRNAs. Required for the assembly of the U4/U6-U5 tri-snRNP complex. |
| **PRPF39** | Pre-MRNA Processing Factor 39 | Involved in pre-mRNA splicing. |
| **PRPF40A** | PRP40 pre-mRNA processing factor 40 homolog A | May be involved in pre-mRNA splicing through interactions with SF1 (splicing factor 1) (85). |
| **PTBP1** | polypyrimidine tract binding protein 1 | Plays a role in pre-mRNA splicing and in the regulation of alternative splicing events. May promote RNA looping when bound to two separate polypyrimidine tracts of introns in the same pre-mRNA. May promote the binding of U2 snRNP to pre-mRNA. |
| **SCAF8** | SR-Related CTD Associated Factor 8 | Identified in a complex with CDC5L and other spliceosomal proteins. |
| **SF3A3** | splicing factor 3a subunit 3 | Involved in pre-mRNA splicing as a component of the splicing factor SF3A complex that contributes to the assembly of the 17S U2 snRNP, and the subsequent assembly of the pre-spliceosome 'E' complex and the pre-catalytic spliceosome 'A' complex. Involved in pre-mRNA splicing as a component of pre-catalytic spliceosome 'B' complexes. |
| **SMNDC1** | Survival Motor Neuron Domain Containing 1 | Necessary for spliceosome assembly. |
| **SNRNP40** | small nuclear ribonucleoprotein U5 subunit 40 | Required for pre-mRNA splicing as a component of the U5 small nuclear ribonucleoprotein (snRNP) complex and the U4/U6-U5 tri-snRNP complex. |
| **SNRNP200** | small nuclear ribonucleoprotein U5 subunit 200 | Plays role in pre-mRNA splicing as core component of precatalytic, catalytic and postcatalytic spliceosomal complexes. Involved in spliceosome assembly, activation and disassembly. Catalyzes the ATP-dependent unwinding of U4/U6 RNA duplexes, an essential step in the assembly of a catalytically active spliceosome. |
| **SRSF2** | Serine and Arginine Rich Splicing Factor 2 | Required for formation of the earliest ATP-dependent splicing complex and interacts with spliceosomal components bound to both the 5'- and 3'-splice sites during spliceosome assembly. It also is required for ATP-dependent interactions of both U1 and U2 snRNPs with pre-mRNA. Interacts with other spliceosomal components, via the RS domains, to form a bridge between the 5'- and 3'-splice site binding components, U1 snRNP and U2AF. |
| **SRSF11** | Serine and Arginine Rich Splicing Factor 11 | May function in pre-mRNA splicing. Contains an arginine/serine-rich region similar to segments found in pre-mRNA splicing factors. |
| **SUGP1 (SF4)** | SURP and G-patch domain containing 1 | Plays a role in pre-mRNA splicing. Interacts with U2AF2 (86, 87). |
| **THOC3** | THO complex 3 | Required for efficient export of polyadenylated RNA and spliced mRNA. Acts as component of the THO subcomplex of the TREX complex which is thought to couple mRNA transcription, processing and nuclear export, and which specifically associates with spliced mRNA and not unspliced pre-mRNA. |
| **TRA2B** | Transformer 2 Beta Homolog | Sequence-specific RNA-binding protein which participates in the control of pre-mRNA splicing. Can either activate or suppress exon inclusion. Alters pre-mRNA splicing patterns by antagonizing the effects of splicing regulators. |
| **TTF2** | Transcription Termination Factor 2 | Interacts with cell division cycle 5-like, associates with human splicing complexes, and plays a role in pre-mRNA splicing. |
| **U2AF1** | U2 small nuclear RNA auxiliary factor 1 | Plays a critical role in both constitutive and enhancer-dependent splicing by mediating protein-protein interactions and protein-RNA interactions required for accurate 3'-splice site selection. Recruits U2 snRNP to the branch point. Directly mediates interactions between U2AF2 and proteins bound to the enhancers and thus may function as a bridge between U2AF2 and the enhancer complex to recruit it to the adjacent intron. |
| **USP39** | Ubiquitin Specific Peptidase 39 | Plays a role in pre-mRNA splicing as a component of the U4/U6-U5 tri-snRNP. |
| **WTAP** | Wilms tumor 1 associated protein | Associated component of the WMM complex, a complex that mediates N6-methyladenosine (m6A) methylation of RNAs, a modification that plays a role in the efficiency of mRNA splicing and RNA processing. Acts as a mRNA splicing regulator. |
| **ZCCHC8** | Zinc Finger CCHC-Type Containing 8 | An accessory factor to the nuclear RNA exosome complex. This complex is also thought to recruit the exosome to degrade intronic RNAs via its interaction with both the exosome and the spliceosome. |
| **ZNF326** | Zinc Finger Protein 326 | Core component of the DBIRD complex, a multiprotein complex that acts at the interface between core mRNP particles and RNA polymerase II (RNAPII) and integrates transcript elongation with the regulation of alternative splicing. |

Note: PPP1R9B was identified but not included in this list as a clear function in splicing was not identified.
