# Supplementary material for: Comparative analysis of retroviral Gag-host cell interactions: focus on the nuclear interactome
Source: Retrovirology. 2024 Jun 19;21:13. doi: 10.1186/s12977-024-00645-y (PMC11186191; doi:10.1186/s12977-024-00645-y)
Supplement: Supplementary file 12 — Supplementary Material 12: Table S8. Top 10 DAVID biological processes of nuclear proteins identified in Ritchie et al. [35]. [file 12977_2024_645_MOESM12_ESM.docx]

**Table S13.** Names and functions of the proteins identified in the HIV-1 proteomics list under GO:0006366~ transcription from RNA polymerase II promoter.

| **Symbol** | **Description** | **Function related to Transcription [mostly summarized from Genecards (62)]** | **Frequency**  **(# of publications)** |
| --- | --- | --- | --- |
| **ALYREF** | Aly/REF Export Factor | Component of the TREX complex which is thought to couple mRNA transcription, processing and nuclear export, and specifically associates with spliced mRNA and not with unspliced pre-mRNA. TREX is recruited to spliced mRNAs by a transcription-independent mechanism, binds to mRNA upstream of the exon-junction complex (EJC) and is recruited in a splicing- and cap-dependent manner to a region near the 5' end of the mRNA where it functions in mRNA export to the cytoplasm. Involved in transcription elongation and genome stability. Acts as chaperone and promotes the dimerization of transcription factors containing basic leucine zipper (bZIP) domains and thereby promotes transcriptional activation. | 2 |
| **ARID4A** | AT-rich interactive domain-containing protein 4A | DNA-binding protein which modulates activity of several transcription factors. May function as part of an mSin3A repressor complex. | 0 |
| **ASH2L** | ASH2 Like, Histone Lysine Methyltransferase Complex Subunit | Component or associated component of some histone methyltransferase complexes which regulates transcription through recruitment of those complexes to gene promoters. | 0 |
| **ASUN**  **(INTS13)** | asunder, spermatogenesis regulator | A probable component of the Integrator (INT) complex – involved in the small nuclear RNAs (snRNA) U1 and U2 transcription and 3’ processing. The INT complex is associated with the C-terminal domain (CTD) of RNA polymerase II largest subunit (POLR2A) and is recruited to the U1 and U2 snRNAs genes. | 1 |
| **BRMS1** | breast cancer metastasis suppressor 1 | Transcriptional repressor. Down-regulates NF-κB transcriptional activation. Promotes HDAC1 binding to promoter regions. | 0 |
| **C1QBP** | Complement component 1 Q subcomponent-binding protein, mitochondrial | May be involved in regulation of FOXC1 transcriptional activity and NFY/CCAAT-binding factor complex-mediated transcription. | 1 |
| **CDK12** | cyclin dependent kinase 12 | Cyclin-dependent kinase that phosphorylates the C-terminal domain (CTD) of the large subunit of RNA polymerase II (POLR2A), thereby acting as a key regulator of transcription elongation. Regulates the expression of genes involved in DNA repair and is required for the maintenance of genomic stability. | 1 |
| **CDKN2A** | cyclin dependent kinase inhibitor 2A  ([Tumor suppressor ARF](https://www.ebi.ac.uk/interpro/entry/IPR010868)) | Has an isoform (ARF) that binds to BCL6 and down-regulates BCL6-induced transcriptional repression. Binds to E2F1 and MYC and blocks their transcriptional activator activity but has no effect on MYC transcriptional repression. Binds to TOP1/TOPOI and stimulates its activity. This complex binds to rRNA gene promoters and may play a role in rRNA transcription and/or maturation. | 0 |
| **CGGBP1** | CGG Triplet Repeat Binding Protein 1 | Binds to nonmethylated 5'-d(CGG)(n)-3' trinucleotide repeats in the FMR1 promoter. May play a role in regulating FMR1 promoter. | 0 |
| **CHTOP** | Chromatin Target of PRMT1 | Required for effective mRNA nuclear export and is a component of the TREX complex (see above). | 2 |
| **CIC** | capicua transcriptional repressor (protein capicua homolog) | Transcriptional repressor which plays a role in development of the central nervous system (CNS). | 0 |
| **CLP1** | Cleavage Factor Polyribonucleotide Kinase Subunit 1 | Member of the tRNA splicing complex. May be involved in the termination of RNA polymerase II transcription (69). | 0 |
| **CPSF3** | cleavage and polyadenylation specific factor 3 | Member of the cleavage and polyadenylation specificity factor (CPSF) complex. May be involved in the termination of RNA polymerase II transcription (69). | 0 |
| **CPSF3L**  **(INTS11)** | cleavage and polyadenylation specific factor 3-like  (Integrator Complex Subunit 11) | Catalytic component of the Integrator (INT) complex (see above). | 0 |
| **CPSF7** | Cleavage And Polyadenylation Specific Factor 7 | Member of the CPSF complex. May be involved in the termination of RNA polymerase II transcription (69). | 0 |
| **CREBBP** | CREB binding protein | Acetylates histones, giving a specific tag for transcriptional activation. | 0 |
| **CTNNB1** | Catenin beta 1 | In the presence of Wnt ligand, CTNNB1 accumulates in the nucleus, where it acts as a coactivator for transcription factors of the TCF/LEF family, leading to activate Wnt responsive genes. | 1 |
| **DDX20** | DEAD-Box Helicase 20 | Represses Egr2-mediated transcriptional activation (88). | 1 |
| **DNAJA3** | DnaJ heat shock protein family (Hsp40) member A3 | Can modulate IFN-gamma-mediated transcriptional activity. | 1 |
| **DNMT1** | DNA methyltransferase | Mediates transcriptional repression by direct binding to HDAC2. In association with DNMT3B and via the recruitment of CTCFL/BORIS, involved in activation of BAG1 gene expression by modulating dimethylation of promoter histone H3 at H3K4 and H3K9. | 1 |
| **EHMT2** | Euchromatic Histone Lysine Methyltransferase 2 | Histone methyltransferase that specifically mono- and dimethylates 'Lys-9' of histone H3 (H3K9me1 and H3K9me2, respectively) in euchromatin. H3K9me represents a specific tag for epigenetic transcriptional repression by recruiting HP1 proteins to methylated histones. | 0 |
| **FIP1L1** | Factor Interacting With PAPOLA And CPSF1 | Member of the CPSF complex. May be involved in RNA polymerase II transcription termination (69). | 1 |
| **FOXP1** | forkhead box P1 | Transcriptional repressor. | 0 |
| **GATAD2A** | GATA zinc finger domain containing 2A | Transcriptional repressor. Enhances MBD2-mediated repression. Efficient repression requires the presence of GATAD2B. | 0 |
| **GTF2H1** | general transcription factor IIH subunit 1 | Component of the general transcription and DNA repair factor IIH (TFIIH) core complex, which is involved in general and transcription-coupled nucleotide excision repair (NER) of damaged DNA and, when complexed to CAK, in RNA transcription by RNA polymerase II. In transcription, TFIIH has an essential role in transcription initiation. | 0 |
| **GTF2H3** | general transcription factor IIH subunit 3 | See above. | 0 |
| **GTF2H4** | general transcription factor IIH subunit 4 | See above. | 0 |
| **HDAC3** | histone deacetylase 3 | Responsible for the deacetylation of lysine residues on the N-terminal part of the core histones (H2A, H2B, H3 and H4), and some other non-histone substrates. Histone deacetylation gives a tag for epigenetic repression and plays an important role in transcriptional regulation, cell cycle progression and developmental events. | 0 |
| **HIST1H1E** | H1.4 Linker Histone, Cluster Member | Histone H1 protein binds to linker DNA between nucleosomes forming the macromolecular structure known as the chromatin fiber. Histones H1 are necessary for the condensation of nucleosome chains into higher-order structured fibers. Acts also as a regulator of individual gene transcription through chromatin remodeling, nucleosome spacing and DNA methylation. | 2 |
| **HIST2H3A** | H3 Clustered Histone 15 | Core component of nucleosome. Nucleosomes wrap and compact DNA into chromatin, limiting DNA accessibility to the cellular machineries which require DNA as a template. Histones thereby play a central role in transcription regulation, DNA repair, DNA replication and chromosomal stability. DNA accessibility is regulated via a complex set of post-translational modifications of histones, also called histone code, and nucleosome remodeling. | 0 |
| **ILK** | Integrin Linked Kinase | The kinase activity regulates key signaling pathways, leading to the stimulation of downstream effector kinases and transcription factors (89). | 0 |
| **INO80** | INO80 Complex ATPase Subunit | ATPase component of the chromatin remodeling INO80 complex which is involved in transcriptional regulation, DNA replication and DNA repair. | 0 |
| **INTS9** | integrator complex subunit 9 | Component of the Integrator (INT) complex (see above). | 1 |
| **LEO1** | LEO1 homolog, Paf1/RNA polymerase II complex component | Component of the PAF1 complex (PAF1C) which has multiple functions during transcription by RNA polymerase II and is implicated in regulation of development and maintenance of embryonic stem cell pluripotency. PAF1C associates with RNA polymerase II through interaction with POLR2A CTD non-phosphorylated and 'Ser-2'- and 'Ser-5'-phosphorylated forms and is involved in transcriptional elongation. | 0 |
| **MBD2** | Methyl-CpG Binding Domain Protein 2 | Recruits histone deacetylases and DNA methyltransferases. Acts as transcriptional repressor and plays a role in gene silencing. | 0 |
| **MECP2** | Methyl-CpG Binding Protein 2 | Mediates transcriptional repression through interaction with histone deacetylase and the corepressor SIN3A. | 0 |
| **MED13** | mediator complex subunit 13 | Component of the Mediator complex, a coactivator involved in the regulated transcription of nearly all RNA polymerase II-dependent genes. Mediator functions as a bridge to convey information from gene-specific regulatory proteins to the basal RNA polymerase II transcription machinery. Mediator is recruited to promoters by direct interactions with regulatory proteins and serves as a scaffold for the assembly of a functional preinitiation complex with RNA polymerase II and the general transcription factors. | 0 |
| **MED15** | mediator complex subunit 15 | Component of the Mediator complex (see above). Required for cholesterol-dependent gene regulation. | 0 |
| **MED21** | mediator complex subunit 21 | Component of the Mediator complex (see above). | 0 |
| **MED23** | Mediator Complex Subunit 23 | Required for transcriptional activation subsequent to the assembly of the pre-initiation complex. Component of the Mediator complex (see above). | 0 |
| **MED26** | Mediator Complex Subunit 26 | Component of the Mediator complex (see above). | 1 |
| **MEIS2** | Meis Homeobox 2 | Involved in transcriptional regulation. | 1 |
| **MINA**  **(RIOX2)** | MYC induced nuclear antigen  (Ribosomal Oxygenase 2) | Oxygenase that can act as both a histone lysine demethylase and a ribosomal histidine hydroxylase. Is involved in the demethylation of trimethylated 'Lys-9' on histone H3 (H3K9me3), leading to an increase in ribosomal RNA expression. | 1 |
| **MTDH** | Metadherin | Downregulates SLC1A2/EAAT2 promoter activity when expressed ectopically. Activates the nuclear factor kappa-B (NF-kappa-B) transcription factor. | 4 |
| **NCOR1** | Nuclear Receptor Corepressor 1 | Mediates transcriptional repression by certain nuclear receptors. Part of a complex which promotes histone deacetylation and the formation of repressive chromatin structures which may impede the access of basal transcription factors. | 0 |
| **NFYA** | Nuclear Transcription Factor Y Subunit Alpha | Component of the sequence-specific heterotrimeric transcription factor (NF-Y) which specifically recognizes a 5'-CCAAT-3' box motif found in the promoters of its target genes. NF-Y can function as both an activator and a repressor, depending on its interacting cofactors. | 0 |
| **OGT** | O-linked N-acetylglucosamine (GlcNAc) transferase | Component of a THAP1/THAP3-HCFC1-OGT complex that is required for the regulation of the transcriptional activity of RRM1. Plays a key role in chromatin structure by mediating O-GlcNAcylation of 'Ser-112' of histone H2B: recruited to CpG-rich transcription start sites of active genes via its interaction with TET proteins (TET1, TET2 or TET3). As part of the NSL complex indirectly involved in acetylation of nucleosomal histone H4 on several lysine residues. | 0 |
| **PABPN1** | poly(A) binding protein nuclear 1 | Cooperates with SKIP to synergistically activate E-box-mediated transcription through MYOD1 and may regulate the expression of muscle-specific genes. | 1 |
| **PAXBP1** | PAX3 And PAX7 Binding Protein 1 | Adapter protein linking the transcription factors PAX3 and PAX7 to the histone methylation machinery. Associates with a histone methyltransferase complex that specifically mediates dimethylation and trimethylation of 'Lys-4' of histone H3. | 1 |
| **PBX1** | PBX homeobox 1 | Acts as a transcriptional activator of PF4 in complex with MEIS1. PDX1:PBX1b:MEIS2b complex is involved in the transcriptional activation of the ELA1 enhancer. Probably in complex with MEIS2, is involved in transcriptional regulation by KLF4. Acts as a transcriptional activator of NKX2-5 and a transcriptional repressor of CDKN2B. | 0 |
| **PCBP1** | poly(rC) binding protein 1 | Involved in the transcriptional activation of BRCA1 promoter (90), eukaryotic translation initiation factor 4E (91), and the mouse mu opioid receptor (92). | 1 |
| **PCF11** | PCF11 Cleavage and Polyadenylation Factor Subunit | The encoded protein is necessary for efficient Pol II transcription termination and may be involved in degradation of the 3' product of polyA site cleavage. | 0 |
| **POLDIP3** | DNA polymerase delta interacting protein 3 | May be involved in RNA polymerase II transcription termination (69). | 0 |
| **POLR2B** | RNA Polymerase II Subunit B | Second largest component of RNA polymerase II. Proposed to contribute to the polymerase catalytic activity and forms the polymerase active center together with the largest subunit. | 0 |
| **POLR2E** | RNA polymerase II subunit E | Common component of RNA polymerases I, II and III. In Pol II, POLR2E/RPB5 is part of the lower jaw surrounding the central large cleft and thought to grab the incoming DNA template. | 0 |
| **PSMC3** | proteasome 26S subunit, ATPase 3 | One of the ATPase subunits, a member of the triple-A family of ATPases that have chaperone-like activity. This subunit may compete with PSMC2 for binding to the HIV tat protein to regulate the interaction between the viral protein and the transcription complex. | 1 |
| **RAD21** | RAD21 cohesin complex component | In interphase, cohesins may function in the control of gene expression by binding to numerous sites within the genome. May control RUNX1 gene expression. Binds to and represses APOB gene promoter. | 0 |
| **RBM10** | RNA binding motif protein 10 | Component of a large chromatin remodeling complex, composed of MYSM1, PCAF, RBM10 and KIF11/TRIP5. | 2 |
| **RBM14** | RNA Binding Motif Protein 14 | Isoform 1 may function as a nuclear receptor coactivator, enhancing transcription through other coactivators such as NCOA6 and CITED1. Isoform 2, functions as a transcriptional repressor, modulating transcriptional activities of coactivators including isoform 1, NCOA6 and CITED1. | 3 |
| **RFC1** | replication factor C subunit 1 | Could play a role in DNA transcription regulation as well as DNA replication and/or repair. Interacts with C-terminus of PCNA. | 2 |
| **RNMT** | RNA guanine-7 methyltransferase | Interacts with elongating form of polymerase II and RNGTT | 0 |
| **SAFB** | Scaffold Attachment Factor B | Binds to scaffold/matrix attachment region (S/MAR) DNA and forms a molecular assembly point to allow the formation of a 'transcriptosomal' complex (consisting of SR proteins and RNA polymerase II) coupling transcription and RNA processing. | 0 |
| **SETD2** | SET Domain Containing 2, Histone Lysine Methyltransferase | Histone methyltransferase that specifically trimethylates 'Lys-36' of histone H3 (H3K36me3), a specific tag for epigenetic transcriptional activation. Plays a role in chromatin structure modulation during elongation by coordinating recruitment of the FACT complex and by interacting with hyperphosphorylated POLR2A. | 0 |
| **SMAD2** | SMAD family member 2 | Receptor-regulated SMAD (R-SMAD) that is an intracellular signal transducer and transcriptional modulator activated by TGF-beta (transforming growth factor) and activin type 1 receptor kinases. The SMAD2/SMAD4 complex functions to activate transcription. | 0 |
| **SMAD5** | SMAD Family Member 5 | Transcriptional modulator activated by BMP (bone morphogenetic proteins) type 1 receptor kinase. | 0 |
| **SMARCA4** | SWI/SNF Related, Matrix Associated, Actin Dependent Regulator Of Chromatin, Subfamily A, Member 4 | Involved in transcriptional activation and repression of select genes by chromatin remodeling (alteration of DNA-nucleosome topology). Component of SWI/SNF chromatin remodeling complexes that carry out key enzymatic activities, changing chromatin structure by altering DNA-histone contacts within a nucleosome in an ATP-dependent manner. | 1 |
| **SMARCB1** | SWI/SNF related, matrix associated, actin dependent regulator of chromatin, subfamily b, member 1 | Core component of the BAF (hSWI/SNF) complex. This ATP-dependent chromatin-remodeling complex. Involved in activation of CSF1 promoter. | 0 |
| **SMARCC2** | SWI/SNF related, matrix associated, actin dependent regulator of chromatin subfamily c member 2 | Involved in transcriptional activation and repression of select genes by chromatin remodeling (alteration of DNA-nucleosome topology). Component of SWI/SNF chromatin remodeling complexes (see above). | 1 |
| **SQSTM1** | sequestosome 1 | Regulates activation of the NF-kB signaling pathway. The protein functions as a scaffolding/adaptor protein in concert with TNF receptor-associated factor 6 to mediate activation of NF-kB in response to upstream signals. | 0 |
| **SRRM1** | serine and arginine repetitive matrix 1 | Has a role in splicing. May be involved in RNA polymerase II transcription termination (69). | 0 |
| **SRSF3**  **(SRp20)** | serine and arginine rich splicing factor 3 | Has a role in splicing. May be involved in RNA polymerase II transcription termination (69). | 3 |
| **SRSF7**  **(9G8)** | serine and arginine rich splicing factor 7 | Has a role in splicing. May be involved in RNA polymerase II transcription termination (69). | 3 |
| **SUDS3** | SDS3 Homolog, SIN3A Corepressor Complex Component | Regulatory protein which represses transcription and augments histone deacetylase activity of HDAC1. May have a potential role in tumor suppressor pathways through regulation of apoptosis. May function in the assembly and/or enzymatic activity of the mSin3A corepressor complex or in mediating interactions between the complex and other regulatory complexes. | 0 |
| **SUPT16H** | SPT16 Homolog, Facilitates Chromatin Remodeling Subunit | Component of the FACT complex, a general chromatin factor that acts to reorganize nucleosomes. During transcription elongation the FACT complex acts as a histone chaperone that both destabilizes and restores nucleosomal structure. It facilitates the passage of RNA polymerase II and transcription by promoting the dissociation of one histone H2A-H2B dimer from the nucleosome, then subsequently promotes the reestablishment of the nucleosome following the passage of RNA polymerase II. | 1 |
| **SUZ12** | SUZ12 Polycomb Repressive Complex 2 Subunit | Polycomb group (PcG) protein. Component of the PRC2/EED-EZH2 complex, which methylates 'Lys-9' (H3K9me) and 'Lys-27' (H3K27me) of histone H3, leading to transcriptional repression of the affected target gene. | 0 |
| **TARDBP** | TAR DNA Binding Protein | Transcriptional repressor that binds to chromosomally integrated TAR DNA and represses HIV-1 transcription. | 0 |
| **TCEB1**  **(ELOC)** | transcription elongation factor B subunit 1 | A general transcription elongation factor that increases the RNA polymerase II transcription elongation past template-encoded arresting sites. | 1 |
| **TGFB1I1** | transforming growth factor beta 1 induced transcript 1 | In the nucleus, functions as a nuclear receptor coactivator regulating glucocorticoid, androgen, mineralocorticoid and progesterone receptor transcriptional activity. | 0 |
| **THOC3** | THO complex 3 | Acts as component of the THO subcomplex of the TREX complex which is thought to couple mRNA transcription, processing and nuclear export, and which specifically associates with spliced mRNA and not unspliced pre-mRNA. | 0 |
| **TRIM33** | tripartite motif containing 33 | May act as a transcriptional repressor. Inhibits the transcriptional response to TGF-beta/BMP signaling cascade. | 0 |
| **TRPS1** | Transcriptional Repressor GATA Binding 1 | Transcriptional repressor. | 0 |
| **U2AF1** | U2 small nuclear RNA auxiliary factor 1 | Has a role in splicing. May be involved in RNA polymerase II transcription termination (69). | 1 |
| **UBE2I** | Ubiquitin Conjugating Enzyme E2 I | Interacts with various transcription factors such as TFAP2A, TFAP2B, TFAP2C, AR, ETS1 and SOX4. | 0 |
| **VWA9**  **(INTS14)** | von Willebrand factor A domain containing 9 | Probable component of the Integrator (INT) complex (see above). | 0 |
| **WDR5** | WD repeat domain 5 | Contributes to histone modification. | 1 |
| **WDR61** | WD repeat domain 61 | Component of the PAF1 complex (PAF1C) which has multiple functions during transcription by RNA polymerase II (see above). Required for Hox gene transcription. | 0 |
| **ZBTB7B** | Zinc Finger And BTB Domain Containing 7B | Transcription regulator that acts as a key regulator of lineage commitment of immature T-cell precursors. Transcriptional repressor of the collagen COL1A1 and COL1A2 genes. May also function as a repressor of fibronectin and possibly other extracellular matrix genes. | 0 |
| **ZEB1** | Zinc Finger E-Box Binding Homeobox 1 | Acts as a transcriptional repressor. | 0 |
