# Supplementary material for: Comparative analysis of retroviral Gag-host cell interactions: focus on the nuclear interactome
Source: Retrovirology. 2024 Jun 19;21:13. doi: 10.1186/s12977-024-00645-y (PMC11186191; doi:10.1186/s12977-024-00645-y)
Supplement: Supplementary file 15 — Supplementary Material 15: Table S11. Names and functions of the proteins identified in the RSV proteomics list under GO:0006366 ~ transcription from RNA polymerase II promoter. [file 12977_2024_645_MOESM15_ESM.docx]

**Table S11.** Names and functions of the proteins identified in the RSV proteomics list under GO:0006366~ transcription from RNA polymerase II promoter.

| **Symbol** | **Description** | **Function related to Transcription [mostly summarized from Genecards (60)]** |
| --- | --- | --- |
| **ARID4A** | AT-rich interactive domain-containing protein 4A | DNA-binding protein which modulates activity of several transcription factors. May function as part of an mSin3A repressor complex. |
| **ATXN1L** | Ataxin-1-like | Chromatin-binding factor that repress Notch signaling in the absence of Notch intracellular domain by acting as a CBF1 corepressor. Binds to the HEY promoter and might assist, along with NCOR2, RBPJ-mediated repression. |
| **CCNK** | Cyclin-K | Regulatory subunit of cyclin-dependent kinases that mediates activation of target kinases. Plays a role in transcriptional regulation via its role in regulating the phosphorylation of the C-terminal domain (CTD) of the large subunit of RNA polymerase II (POLR2A). |
| **CDK13** | Cyclin Dependent Kinase 13 | Hyperphosphorylates the C-terminal heptapeptide repeat domain (CTD) of the largest RNA polymerase II subunit RPB1, thereby acting as a key regulator of transcription elongation. |
| **CHD7** | Chromodomain Helicase DNA Binding Protein 7 | May interact with CTCF (see below). Is a member of the chromodomain helicase DNA-binding family of ATP-dependent chromatin remodeling enzymes (81). |
| **CHTOP** | Chromatin Target of PRMT1 | Required for effective mRNA nuclear export and is a component of the TREX complex which is thought to couple mRNA transcription, processing and nuclear export, and specifically associates with spliced mRNA and not with unspliced pre-mRNA. |
| **CNOT7** | CCR4-NOT transcription complex subunit 7 | Catalytic component of the CCR4-NOT complex which is one of the major cellular mRNA deadenylases and is linked to various cellular processes including bulk mRNA degradation, miRNA-mediated repression, translational repression during translational initiation and general transcription regulation. |
| **CTCF** | CCCTC-Binding Factor | Chromatin binding factor that binds to DNA sequence specific sites. Involved in transcriptional regulation by binding to chromatin insulators and preventing interaction between promoter and nearby enhancers and silencers. Inversely, binding to target sites is prevented by CpG methylation. Plays an important role in chromatin remodeling preventing spreading of CpG methylation and maintaining methylation-free zones. |
| **CUX1** | Cut Like Homeobox 1 | Transcription factor involved in the control of neuronal differentiation in the brain. It probably has a broad role in mammalian development as a repressor of developmentally regulated gene expression. May act by preventing binding of positively-activating CCAAT factors to promoters. |
| **EIF4A3** | Eukaryotic Translation Initiation Factor 4A3 | Involved in splicing. May be involved in RNA polymerase II transcription termination (69). |
| **ETV6** | ETS Variant Transcription Factor 6 | Transcriptional repressor; binds to the DNA sequence 5'-CCGGAAGT-3'. |
| **FOXK2** | Forkhead Box K2 | Transcriptional regulator involved in different processes such as glucose metabolism, aerobic glycolysis and autophagy. Recognizes and binds the forkhead DNA sequence motif (5'-GTAAACA-3') and can both act as a transcription activator or repressor, depending on the context. |
| **FOXP1** | Forkhead Box P1 | Transcriptional repressor. |
| **HCFC2** | Host Cell Factor C2 | Component of the MLL1/MLL complex, a multiprotein complex that mediates both methylation of 'Lys-4' of histone H3 (H3K4me) complex, a specific tag for epigenetic transcriptional activation. |
| **HNRNPK** | heterogeneous nuclear ribonucleoprotein H3 | Plays an important role in p53/TP53 response to DNA damage, acting at the level of both transcription activation and repression. |
| **INTS3** | Integrator complex subunit 3 | Component of the Integrator (INT) complex. The Integrator complex is involved in the small nuclear RNAs (snRNA) U1 and U2 transcription and in their 3'-box-dependent processing. The Integrator complex is associated with the C-terminal domain (CTD) of RNA polymerase II largest subunit (POLR2A). |
| **INTS5** | Integrator Complex Subunit 5 | Component of the Integrator (INT) complex (see above). |
| **JUN** | Jun Proto-Oncogene, AP-1 Transcription Factor Subunit | Transcription factor that recognizes and binds to the enhancer heptamer motif 5'-TGA[CG]TCA-3'. |
| **LEF1** | Lymphoid Enhancer Binding Factor 1 | Transcription factor that binds DNA in a sequence-specific manner. Participates in the Wnt signaling pathway. |
| **LEO1** | LEO1 Homolog, Paf1/RNA Polymerase II Complex Component | Component of the PAF1 complex (PAF1C) which has multiple functions during transcription by RNA polymerase II and is implicated in regulation of development and maintenance of embryonic stem cell pluripotency. PAF1C associates with RNA polymerase II through interaction with POLR2A CTD non-phosphorylated and 'Ser-2'- and 'Ser-5'-phosphorylated forms and is involved in transcriptional elongation. |
| **MAGOH** | Mago Homolog, Exon Junction Complex Subunit | Involved in splicing. May be involved in RNA polymerase II transcription termination (69). |
| **MBD3** | Methyl-CpG-binding domain protein 3 | Acts as transcriptional repressor and plays a role in gene silencing. |
| **MED6** | Mediator of RNA polymerase II transcription subunit 6 | Component of the Mediator complex, a coactivator involved in the regulated transcription of nearly all RNA polymerase II-dependent genes. Mediator functions as a bridge to convey information from gene-specific regulatory proteins to the basal RNA polymerase II transcription machinery. Mediator is recruited to promoters by direct interactions with regulatory proteins and serves as a scaffold for the assembly of a functional preinitiation complex with RNA polymerase II and the general transcription factors. |
| **MED24** | Mediator of RNA polymerase II transcription subunit 24 | Component of the Mediator complex (see above). |
| **MED26** | Mediator of RNA polymerase II transcription subunit 26 | Component of the Mediator complex (see above). |
| **MED30** | Mediator of RNA polymerase II transcription subunit 30 | Component of the Mediator complex (see above). |
| **NOC2L** | NOC2 Like Nucleolar Associated Transcriptional Repressor | Acts as an inhibitor of histone acetyltransferase activity; prevents acetylation of all core histones by the EP300/p300 histone acetyltransferase at p53/TP53-regulated target promoters in a histone deacetylases (HDAC)-independent manner. Acts as a transcription corepressor of p53/TP53- and TP63-mediated transactivation of the p21/CDKN1A promoter. |
| **OGT** | UDP-N-acetylglucosamine--peptide N-acetylglucosaminyltransferase 110 kDa subunit | Component of a THAP1/THAP3-HCFC1-OGT complex that is required for the regulation of the transcriptional activity of RRM1. Plays a key role in chromatin structure by mediating O-GlcNAcylation of 'Ser-112' of histone H2B: recruited to CpG-rich transcription start sites of active genes via its interaction with TET proteins (TET1, TET2 or TET3). As part of the NSL complex indirectly involved in acetylation of nucleosomal histone H4 on several lysine residues. |
| **PLK1** | Polo Like Kinase 1 | Phosphorylates FOXM1, a key mitotic transcription regulator, leading to enhance FOXM1 transcriptional activity. Phosphorylates the transactivation domain of the transcription factor p73/TP73, leading to inhibit p73/TP73-mediated transcriptional activation and pro-apoptotic functions. |
| **POLR2B** | RNA Polymerase II Subunit B | Second largest component of RNA polymerase II. Proposed to contribute to the polymerase catalytic activity and forms the polymerase active center together with the largest subunit. |
| **RAD21** | RAD21 Cohesin Complex Component | In interphase, cohesins may function in the control of gene expression by binding to numerous sites within the genome. May control RUNX1 gene expression. Binds to and represses APOB gene promoter. |
| **RFC1** | Replication Factor C Subunit 1 | Could play a role in DNA transcription regulation as well as DNA replication and/or repair. Can bind single- or double-stranded DNA. Interacts with C-terminus of PCNA. 5' phosphate residue is required for binding of the N-terminal DNA-binding domain to duplex DNA, suggesting a role in recognition of non-primer template DNA structures during replication and/or repair. |
| **RPRD1B** | Regulation of nuclear pre-mRNA domain-containing protein 1B | Interacts with phosphorylated C-terminal heptapeptide repeat domain (CTD) of the largest RNA polymerase II subunit POLR2A, and participates in dephosphorylation of the CTD by RPAP2. Prevents RNA polymerase II from reading through the 3' end termination site and may allow it to be recruited back to the promoter through promotion of the formation of a chromatin loop. |
| **SAFB** | Scaffold attachment factor B1 | Binds to scaffold/matrix attachment region (S/MAR) DNA and forms a molecular assembly point to allow the formation of a 'transcriptosomal' complex (consisting of SR proteins and RNA polymerase II) coupling transcription and RNA processing. |
| **SAP30** | Sin3A Associated Protein 30 | Involved in the functional recruitment of the Sin3-histone deacetylase complex (HDAC) to a specific subset of N-CoR corepressor complexes. Active in deacetylating core histone octamers (when in a complex) but inactive in deacetylating nucleosomal histones. |
| **SMAD2** | SMAD Family Member 2 | Receptor-regulated SMAD (R-SMAD) that is an intracellular signal transducer and transcriptional modulator activated by TGF-beta (transforming growth factor) and activin type 1 receptor kinases. The SMAD2/SMAD4 complex functions to activate transcription. |
| **SP3** | Sp3 Transcription Factor | Transcriptional factor that can act as an activator or repressor depending on isoform and/or post-translational modifications. |
| **SRSF2**  **(SC35)** | Serine and Arginine Rich Splicing Factor 2 | A unique SR protein that activates transcription in a position-dependent manner. Binds to promoter-associated small RNAs to mediate transcription pause release (82). |
| **SRSF11** | Serine and Arginine Rich Splicing Factor 11 | Involved in splicing. May be involved in RNA polymerase II transcription termination (69). |
| **STRN3** | Striatin-3 | Binding partner of glucocorticoid receptor (GR) that interferes with GR’s ligand-dependent transactivation capacity. |
| **TAF2** | TATA-Box Binding Protein Associated Factor 2 | Transcription factor TFIID is one of the general factors required for accurate and regulated initiation by RNA polymerase II. TFIID is a multimeric protein complex that plays a central role in mediating promoter responses to various activators and repressors. It requires core promoter-specific cofactors for productive transcription stimulation. TAF2 stabilizes TFIID binding to core promoter. |
| **TAF7** | TATA-Box Binding Protein Associated Factor 7 | Functions as a component of the DNA-binding general transcription factor complex TFIID. |
| **TAF8** | TATA-Box Binding Protein Associated Factor 8 | Functions as a component of the DNA-binding general transcription factor complex TFIID. Mediates both basal and activator-dependent transcription. Required for the integration of TAF10 in the TAF complex. |
| **TAF13** | TATA-Box Binding Protein Associated Factor 13 | Functions as a component of the DNA-binding general transcription factor complex TFIID. |
| **TCEB3**  **(ELOA)** | Elongin A | Also known as elongin, is a general transcription elongation factor that increases the RNA polymerase II transcription elongation past template-encoded arresting sites. Subunit A is transcriptionally active and its transcription activity is strongly enhanced by binding to the dimeric complex of the SIII regulatory subunits B and C (elongin BC complex). |
| **TCF3** | Transcription Factor 3 | Transcriptional regulator. |
| **TCF20** | Transcription Factor 20 | Transcriptional activator that binds to the regulatory region of MMP3 and thereby controls stromelysin expression. It stimulates the activity of various transcriptional activators such as JUN, SP1, PAX6 and ETS1, suggesting a function as a coactivator. |
| **THOC3** | THO complex 3 | Acts as component of the THO subcomplex of the TREX complex which is thought to couple mRNA transcription, processing and nuclear export, and which specifically associates with spliced mRNA and not unspliced pre-mRNA. |
| **TTF2** | Transcription Termination Factor 2 | DsDNA-dependent ATPase which acts as a transcription termination factor by coupling ATP hydrolysis with removal of RNA polymerase II from the DNA template. |
| **U2AF1** | Splicing factor U2AF 35 kDa subunit | Involved in splicing. May be involved in RNA polymerase II transcription termination (69). |
| **VWA9**  **(INTS14)** | von Willebrand factor A domain containing 9  (Integrator Complex Subunit 14) | Probable component of the Integrator (INT) complex, a complex involved in the small nuclear RNAs (snRNA) U1 and U2 transcription and in their 3'-box-dependent processing. |
| **XPO1** | Exportin-1 | Mediates the nuclear export of cellular proteins (cargos) bearing a leucine-rich nuclear export signal (NES) and of RNAs. |
| **ZEB1** | Zinc Finger E-Box Binding Homeobox 1 | Acts as a transcriptional repressor. |
| **ZMYND8** | Protein kinase C-binding protein 1 (Zinc finger MYND domain-containing protein 8) | May act as a transcriptional corepressor for KDM5D. |
