# Supplementary material for: Comparative analysis of retroviral Gag-host cell interactions: focus on the nuclear interactome
Source: Retrovirology. 2024 Jun 19;21:13. doi: 10.1186/s12977-024-00645-y (PMC11186191; doi:10.1186/s12977-024-00645-y)
Supplement: Supplementary file 18 — Supplementary Material 18: Table S14. Names and function of the proteins identified in the HIV-1 proteomics list under the GO:0008380 ~ RNA splicing. [file 12977_2024_645_MOESM18_ESM.docx]

**Table S14.** Names and function of the proteins identified in the HIV-1 proteomics list under the GO:0008380~RNA splicing.

| **Symbol** | **Description** | **Function related to RNA Splicing [mostly summarized from Genecards (60)]** | **Frequency**  **(# of publications)** |
| --- | --- | --- | --- |
| **ALYREF** | Aly/REF Export Factor | Involved in nuclear export of spliced and unspliced mRNAs through specific binding to the m5C by being a component of the TREX complex. The TREX complex functions to couple different steps during mRNA biogenesis, including mRNA transcription, processing, decay, and nuclear export. | 2 |
| **C1QBP** | Complement component 1 Q subcomponent-binding protein, mitochondrial | Involved in regulation of RNA splicing by inhibiting the RNA-binding capacity of SRSF1 and its phosphorylation. Required for the nuclear translocation of splicing factor U2AF1L4. | 1 |
| **CCAR2** | cell cycle and apoptosis regulator 2 | A core component of the DBIRD complex, a multiprotein complex that acts at the interface between core messenger ribonucleoprotein (mRNP) particles and RNA polymerase II and integrates transcript elongation with the regulation of alternative splicing. The DBIRD complex affects local transcript elongation rates and alternative splicing of a large set of exons embedded in (A + T)-rich DNA regions. | 1 |
| **CDC5L** | cell division cycle 5 like | Plays role in precursor mRNA (pre-mRNA) splicing as core component of precatalytic, catalytic and postcatalytic spliceosomal complexes; the PRP19-CDC5L complex. | 3 |
| **CDK12** | cyclin dependent kinase 12 | Required for RNA splicing, possibly by phosphorylating SRSF1/SF2. | 1 |
| **CLP1** | Cleavage Factor Polyribonucleotide Kinase Subunit 1 | Component of the pre-mRNA cleavage complex II (CF-II), which seems to be required for mRNA 3'-end formation. Component of the tRNA splicing endonuclease complex and is implicated in tRNA, mRNA, and siRNA maturation. | 0 |
| **CPSF3** | cleavage and polyadenylation specific factor 3 | Component of the cleavage and polyadenylation specificity factor (CPSF) complex that play a key role in pre-mRNA 3'-end formation and functions as mRNA 3'-end-processing endonuclease. Also involved in the histone 3'-end pre-mRNA processing through U7 small nuclear ribonucleoprotein (snRNP) interactions. | 0 |
| **CPSF4** | cleavage and polyadenylation specific factor 4 | Component of the CPSF complex (see above). Binds RNA polymers with a preference for poly(U). | 0 |
| **CPSF7** | Cleavage and Polyadenylation Specific Factor 7 | Component of the cleavage factor Im (CFIm) complex that functions as an activator of the pre-mRNA 3'-end cleavage and polyadenylation processing. Directly activates the mRNA 3'-processing machinery and binds to polyadenylation signals in RNA substrates. Interacts with the splicing factor U2 snRNP Auxiliary Factor (U2AF) 65 to link the splicing and polyadenylation complexes. | 0 |
| **DDX20** | DEAD-Box Helicase 20 | DEAD box proteins, characterized by the conserved motif Asp-Glu-Ala-Asp (DEAD), are putative RNA helicases. Directly interacts with survival of motor neurons (SMN) complex and may play a catalytic role in the function of the SMN complex on spliceosome RNPs. May also play a role in the metabolism of small nucleolar ribonucleoprotein (snoRNPs). | 1 |
| **DDX46** | DEAD-Box Helicase 46 | DEAD box protein family. Plays an essential role in splicing, either prior to, or during splicing A complex formation. | 1 |
| **DDX47** | DEAD-box helicase 47 | DEAD box protein family. May have a role in rRNA processing and mRNA splicing. Associates with pre-rRNA precursors. | 2 |
| **DNAJC8** | DnaJ Heat Shock Protein Family (Hsp40) Member C8 | Interacts with SRPK1 (see below). | 0 |
| **ELAVL1**  **(HuR)** | ELAV like RNA binding protein 1 | RNA-binding protein that binds to poly-U elements and AU-rich elements in the 3'-UTR region of mRNAs increasing their stability. | 2 |
| **FAM98B** | family with sequence similarity 98 member B | Component of the tRNA-splicing ligase complex (93, 94). | 0 |
| **FIP1L1** | Factor Interacting with PAPOLA And CPSF1 | Component of the CPSF complex. Contributes to poly(A) site recognition and stimulates poly(A) addition. Binds to U-rich RNA sequence elements surrounding the poly(A) site. May act to tether poly(A) polymerase to the CPSF complex. | 1 |
| **HNRNPH3** | heterogeneous nuclear ribonucleoprotein H3 | Involved in the splicing process and participates in early heat shock-induced splicing arrest. | 2 |
| **HNRNPM** | heterogeneous nuclear ribonucleoprotein M | Member of the heterogeneous nuclear ribonucleoproteins (hnRNPs) family. The hnRNPs are RNA binding proteins and they complex with heterogeneous nuclear RNA (hnRNA). These proteins are associated with pre-mRNAs in the nucleus and appear to influence pre-mRNA processing and other aspects of mRNA metabolism and transport. | 3 |
| **HNRNPR** | Heterogeneous Nuclear Ribonucleoprotein R | Component of hnRNPs (see above). A member of the spliceosome C complex. | 2 |
| **HNRNPUL1** | heterogeneous nuclear ribonucleoprotein U like 1 | Plays also a role in mRNA processing and transport. Binds avidly to poly(G) and poly(C) RNA homopolymers *in vitro*. | 2 |
| **LSM3** | LSM3 homolog, U6 small nuclear RNA and mRNA degradation associated | Plays a role in pre-mRNA splicing as component of the U4/U6-U5 tri-snRNP complex that is involved in spliceosome assembly, and as component of the precatalytic spliceosome (spliceosome B complex). | 0 |
| **PABPN1** | poly(A) binding protein nuclear 1 | Involved in the 3'-end formation of pre-mRNAs by the addition of a poly(A) tail of 200-250 nt to the upstream cleavage product.  Promotes intron excision in the context of 3′-end polyadenylation but not when bound to internal A-tracts (95). | 1 |
| **PCBP1** | poly(rC) binding protein 1 | Interaction with SMAD3 regulates the alternative splicing of CD44 (96). | 1 |
| **PCF11** | PCF11 Cleavage and Polyadenylation Factor Subunit | Component of pre-mRNA cleavage complex II. | 0 |
| **PLRG1** | pleiotropic regulator 1 | Component of the PRP19-CDC5L complex that forms an integral part of the spliceosome and is required for activating pre-mRNA splicing. | 3 |
| **POLR2B** | RNA Polymerase II Subunit B | Second largest component of RNA polymerase II | 0 |
| **POLR2E** | RNA polymerase II subunit E | Common component of RNA polymerases I, II and III. | 0 |
| **PPIH** | peptidylprolyl isomerase H | Participates in pre-mRNA splicing. May play a role in the assembly of the U4/U5/U6 tri-snRNP complex of the spliceosome. | 1 |
| **PRPF3** | pre-mRNA processing factor 3 | Plays role in pre-mRNA splicing as component of the U4/U6-U5 tri-snRNP complex in spliceosome complex. Spliceosome B complex. | 4 |
| **PRPF4** | pre-mRNA processing factor 4 | Plays role in pre-mRNA splicing as component of the U4/U6-U5 tri-snRNP complex in spliceosome complex. Spliceosome B complex. | 4 |
| **PRPF38B** | pre-mRNA processing factor 38B | May be required for pre-mRNA splicing. | 0 |
| **PRPF40A** | Pre-MRNA Processing Factor 40 Homolog A | May be involved in pre-mRNA splicing. with SF1 (splicing factor 1) (85). | 0 |
| **PTBP1** | polypyrimidine tract binding protein 1 | Plays a role in pre-mRNA splicing and in the regulation of alternative splicing events. May promote RNA looping when bound to two separate polypyrimidine tracts of introns in the same pre-mRNA. May promote the binding of U2 snRNP to pre-mRNA. | 1 |
| **PUF60** | poly(U) binding splicing factor 60 | DNA- and RNA-binding protein, involved in several nuclear processes such as pre-mRNA splicing, apoptosis and transcription regulation. Promotes splicing of an intron with weak 3'-splice site and pyrimidine tract in a cooperative manner with U2AF2. Modulates alternative splicing of several mRNAs. | 0 |
| **RAVER2** | ribonucleoprotein, PTB binding 2 | Interactions with PTB may be required for role in splicing (97, 98). | 0 |
| **RBM7** | RNA Binding Motif Protein 7 | Interacts with splicing factors, SAP145 and SRp20 (99), as well as U4/U6.U5 tri-snRNP-associated proteins (100). | 1 |
| **RBM10** | RNA binding motif protein 10 | Associates with hnRNP proteins and may be involved in regulating alternative splicing. | 2 |
| **SCAF8** | SR-Related CTD Associated Factor 8 | Identified in a complex with CDC5L and other spliceosomal proteins. | 0 |
| **SMN2** | survival of motor neuron 2, centromeric | The SMN complex plays a catalyst role in the assembly of snRNPs of the spliceosome. Ensures the correct splicing of U12 intron-containing genes. May also play a role in the metabolism of snoRNPs. | 1 |
| **SNRPA** | small nuclear ribonucleoprotein polypeptide A | Component of the spliceosomal U1 snRNP, which is essential for recognition of the pre-mRNA 5' splice-site and the subsequent assembly of the spliceosome. In a snRNP-free form (SF-A) may be involved in coupled pre-mRNA splicing and polyadenylation process. | 2 |
| **SNRPD2** | small nuclear ribonucleoprotein D2 polypeptide | Plays role in pre-mRNA splicing as core component of the SMN-Sm complex that mediates spliceosomal snRNP assembly and as component of the spliceosomal U1, U2, U4 and U5 snRNPs. Component of both the pre-catalytic spliceosome B complex and activated spliceosome C complexes. A component of the minor U12 spliceosome. | 1 |
| **SRPK1** | SRSF Protein Kinase 1 | Serine/arginine-rich protein-specific kinase which specifically phosphorylates its substrates at serine residues located in regions rich in arginine/serine dipeptides, known as RS domains and is involved in the phosphorylation of SR splicing factors and the regulation of splicing. Plays a central role in the regulatory network for splicing, controlling the intranuclear distribution of splicing factors in interphase cells and the reorganization of nuclear speckles during mitosis. | 1 |
| **SRRM1** | serine and arginine repetitive matrix 1 | Part of pre- and post-splicing multiprotein mRNP complexes. Promotes constitutive and exonic splicing enhancer (ESE)-dependent splicing activation by bridging together sequence-specific and basal snRNP factors of the spliceosome. | 0 |
| **SRSF3** | serine and arginine rich splicing factor 3 | Splicing factor that specifically promotes exon-inclusion during alternative splicing. | 3 |
| **SRSF7** | serine and arginine rich splicing factor 7 | Required for pre-mRNA splicing. | 3 |
| **SRSF12** | Serine And Arginine Rich Splicing Factor 12 | Splicing factor that seems to antagonize SR proteins in pre-mRNA splicing regulation. | 0 |
| **TARDBP** | TAR DNA Binding Protein | RNA-binding protein that is involved in various steps of RNA biogenesis and processing. Regulates the splicing of many non-coding and protein-coding RNAs. Regulates mRNA stability. | 0 |
| **THOC3** | THO complex 3z | Required for efficient export of polyadenylated RNA and spliced mRNA. Acts as component of the THO subcomplex of the TREX complex which is thought to couple mRNA transcription, processing and nuclear export, and which specifically associates with spliced mRNA and not unspliced pre-mRNA. | 0 |
| **TRA2B** | Transformer 2 Beta Homolog | Sequence-specific RNA-binding protein which participates in the control of pre-mRNA splicing. Can either activate or suppress exon inclusion. Alters pre-mRNA splicing patterns by antagonizing the effects of splicing regulators. | 2 |
| **U2AF1** | U2 small nuclear RNA auxiliary factor 1 | Plays a critical role in both constitutive and enhancer-dependent splicing by mediating protein-protein interactions and protein-RNA interactions required for accurate 3'-splice site selection. Recruits U2 snRNP to the branch point. Directly mediates interactions between U2AF2 and proteins bound to the enhancers and thus may function as a bridge between U2AF2 and the enhancer complex to recruit it to the adjacent intron. | 1 |
