# Supplementary material for: Comparative analysis of retroviral Gag-host cell interactions: focus on the nuclear interactome
Source: Retrovirology. 2024 Jun 19;21:13. doi: 10.1186/s12977-024-00645-y (PMC11186191; doi:10.1186/s12977-024-00645-y)
Supplement: Supplementary file 19 — Supplementary Material 19: Figure S1. The number of proteins identified in both the RSV and HIV-1 Gag affinity tagged purifications. [file 12977_2024_645_MOESM19_ESM.docx]

**
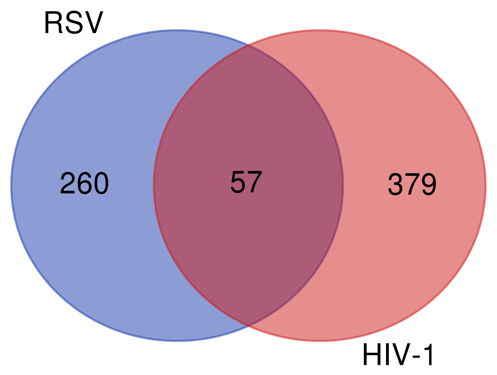
**

**Figure S1.** The number of proteins identified in both the RSV and HIV-1 Gag affinity tagged purifications. 57 proteins were found to be in common between RSV and HIV-1 Gag.
